# ﻿New records of Curculionoidea from Newfoundland and Labrador, with the first records of *Orthochaetessetiger* ([Beck]) (Curculionidae, Curculioninae, Styphlini) for North America

**DOI:** 10.3897/zookeys.1136.91567

**Published:** 2022-12-19

**Authors:** David W. Langor, Robert S. Anderson, Patrice Bouchard, Stephen D. Langor

**Affiliations:** 1 Natural Resources Canada, Canadian Forest Service, 5320 – 122 St. NW, Edmonton, Alberta, T6H 3S5, Canada Natural Resources Canada Edmonton Canada; 2 Canadian Museum of Nature, 1740 Chemin Pink, Gatineau, Quebec, J9J 3N7, Canada Canadian Museum of Nature Quebec Canada; 3 Canadian National Collection of Insects, Arachnids and Nematodes, Agriculture and Agri-Food Canada, 960 Carling Avenue, Ottawa, Ontario, K1A 0C6, Canada Canadian National Collection of Insects, Arachnids and Nematodes, Agriculture and Agri-Food Canada Ottawa Canada; 4 University of Alberta, Department of Occupational Therapy, Corbett Hall, 8205 – 114 St. NW, Edmonton, Alberta, T6G 2G4, Canada University of Alberta Edmonton Canada

**Keywords:** Adventive species, distribution, faunistics, identification key, species list, weevil

## Abstract

Thirty species of Curculionoidea (28 Curculionidae and one each of Brentidae and Nemonychidae) are reported as new records from the Canadian province of Newfoundland and Labrador, most of them from the island of Newfoundland. As well, 13 species of Curculionidae and one of Brentidae previously recorded from Newfoundland are newly reported from Labrador, and one Curculionidae previously recorded from Labrador is newly reported from Newfoundland. The Palearctic species, *Orthochaetessetiger* ([Beck]), is herein reported as a new Canadian and North American record, with specimens documented from Newfoundland and British Columbia. Additions to the primary key for North American weevils are provided to help identify this genus among the North American fauna. Of the species of Curculionoidea previously recorded from the province in published literature, there is uncertain evidence for the occurrence of 14 species in the province as a whole or in the Labrador portion. Seven species are hereby removed from the faunal list for the province. One of those, *Trachodeshispidus* (Linnaeus), is also removed from the Canadian faunal list. The 134 species of Curculionoidea recorded from NL are listed and a brief synopsis of the fauna provided.

## ﻿Introduction

The Curculionidae comprise the largest beetle family in Canada with 839 recorded species (Bousquet et al. 2013; Webster et al. 2016; de Tonnancour et al. 2017; Pentinsaari et al. 2019; Franklin et al. 2021; Thurston et al. 2022). Together the Curculionidae and the related families Anthribidae (22 spp. in Canada), Attelabidae (14), Brachyceridae (18), Brentidae (48), Nemonychidae (8), and Dryophthoridae (27) constitute the superfamily Curculionoidea, commonly known as weevils. Of the 976 species of Curculionoidea in Canada, 111 species (11.4% of the Canadian fauna) have been reported in the province of Newfoundland and Labrador (NL), including 105 species on the island of Newfoundland (NF) and 18 species in Labrador (LB) (Bousquet et al. 2013). Thus, NL is relatively depauperate, and this is attributed to the entire province being glaciated during the Wisconsinan glaciation, that LB is further north and therefore not conducive to high biodiversity, and that NF is separated from the mainland by more than 162 km of ocean. The province’s fauna has also not been particularly well sampled, especially that portion in LB; thus, it is unsurprising that many new records of weevils have been discovered in the province subsequent to publication of the 2013 Canadian beetle checklist (Bousquet et al. 2013). Herein we report on 45 species newly documented from either NF, LB, or the province as a whole. One of these species, *Orthochaetessetiger* ([Beck]), is newly recorded from North America, including Canada, with records from NF and British Columbia.

## ﻿Materials and methods

New records are based on specimens in the six institutional collections with the largest known holdings of NL specimens. Some species identities were verified with DNA barcode data in the Barcode of Life Datasystems (BOLD) database (boldsystems.org), and the 'Sample ID' numbers for barcoded specimens are included under "Specimens examined" for relevant species. More than 6400 specimens were examined and their identity verified, and these represent more than 4000 collection events. A collection event is a unique combination of locality and date where one or more specimens were collected, labelled, and deposited in a public repository. Provincial species records based only on reports in catalogues, e.g., Bousquet et al. (2013), with no accompanying label information and repository of specimens, were given special consideration. For species present in the Maritime provinces of Canada, and for which NL provides suitable host species and climate, the records were retained for now as there seems to be a high likelihood they are valid. Other species were removed from the provincial list. The Curculionoidea classification system used herein follows that of Alonso-Zarazaga et al. (2017). World Flora Online (www.worldfloraonline.org) was used to confirm spellings and authorities for plant species.

### ﻿Specimen repositories and institutional acronyms

**AAFC** Agriculture and Agri-Food Canada collection, St. John’s, Newfoundland and Labrador, Canada;

**CBG** Centre for Biodiversity Genomics, University of Guelph, Guelph, Ontario, Canada;

**CFS**Canadian Forest Service, Atlantic Forestry Centre, Corner Brook, Newfoundland and Labrador, Canada;

**CMNC**Canadian Museum of Nature , Gatineau, Quebec, Canada;

**CNC**Canadian National Collection of Insects, Arachnids and Nematodes, Agriculture and Agri-Food Canada, Ottawa, Ontario, Canada;

**DLC** David Langor Collection of NL Insects, Canadian Forest Service, Edmonton, Alberta, Canada [Note: This collection consists of all the insect material from the former Memorial University of Newfoundland (MUN) Collection. The MUN collection was shipped to David Langor for safe keeping when the last curator (David Larson) retired in 2005 as the institution no longer wished to retain the collection. It is hoped that the collection will be returned to NL in the near future, but a new home has not yet been secured.];

**NFRC** Canadian Forest Service, Northern Forestry Centre, Edmonton, Alberta, Canada;

**RBCM**Royal British Columbia Museum, 675 Belleville St., Victoria, British Columbia, V8W 9W2, Canada.

## ﻿Results

### ﻿BRENTIDAE

**Apioninae: Apionini**

***Betulapionsimilewalshii* (J.B. Smith, 1884)**

This Holarctic species is represented across the Palearctic as the nominotypical subspecies, and in Canada and the USA by *B.similewalshii*. In Canada, it has been reported from British Columbia to NF (Bousquet et al. 2013). Throughout its range the species feeds on catkins of various *Betula* spp. (Betulaceae), primarily *B.papyrifera* Marsh. in North America. Here we report the species for the first time from LB based on five teneral adult specimens.

**Specimens examined**: Labrador: Happy Valley, 10 August 1978, *Betulapapyrifera*, coll. J. Peter Hall (3, DLC, Accession Nos 17-21970 to 17-21972; 2, NFRC, Accession Nos NFRC-2022-07480, NFRC-2022-07481; determined by RSA).

***Perapioncurtirostre* (Germar, 1817)**

This small Palearctic weevil was first recorded from the Maritime provinces of Canada by Majka et al. (2007a) where it is widely distributed. In Nova Scotia it has been associated with *Rumexacetosella* L. and *R.crispus* L. (Polygonaceae). This is a new provincial record for NL where the species is widespread on the island of NF.

**Specimens examined (determined by RSA except where noted)**: Newfoundland: Bunyan’s Cove, 48.3960°N, 54.0129°W, 3 m, 14 August 2008, sweep of flowers, David Langor (3, DLC, Accession Nos 12-19, 17-17621, 17-17622); Cape Anguille, 47.899°N, 59.411°W, 18 m, 22 June 2010, sweep of marsh vegetation, David Langor (2, DLC, Accession Nos 12-24, 17-17623); Chamberlains, 15 October 2001, S. Garland (1, CMN, DLC Accession No. 12-18; 1, NFRC, Accession No. NFRC-2022-07474); Champney’s West, 48.379°N, 53.298°W, 15 August 2014, sweep of vegetation on coastal barrens, David & Matthew Langor (1, DLC, Accession No. 17-17625); Clarenville, 48.173°N, 53.964°W, 22 July 2022, sweep of vegetation, D. Langor (DLC, 2, Accession Nos 17-22051, 17-22062; determined by DWL); Logy Bay, 47.6306°N, 52.6874°W, 29 July 2014, sweep of grass, Mardon Erbland (1, iNaturalist, inaturalist.org/observations/819784); Paddy’s Pond, 26 Sept. 2002 (2, DLC, Accession Nos 12-17, 17-17624; latter with determination confirmed using DNA Barcode – BOLD: CCDB-28535-D06); Pasadena Beach, 49.022°N, 57.608°W, 21 m, 25 June 2010, sweep of vegetation, David Langor (1, DLC, Accession No. 12-23); Searston, 47.8299°N, 59.3064°W, 10 August 2022, sweep of vegetation on disturbed roadside and in field, D. Langor (1, DLC, Accession No. 17-22048; determined by DWL); St. Anthony, 51.365°N, 55.5918°W, 17 August 2022, sweep of vegetation on disturbed open areas, D. Langor (4, DLC, Accession Nos 17-22044 to 17-22047; determined by DWL); St. David’s, 48.205°N, 58.866°W, 10 July, 2008, sweep of fallow field, coll. Goulet, Boudreault and Badiss (2, DLC, Accession Nos 12-20, 17-17618; 1, CMN, DLC Accession No. 17-17619); St. John’s, 47.598°N, 52.713°W, 18 to 22 June 2006, sweep of shrubs and forbs, David Langor (3, NFRC, Accession Nos 2022-07475 to 2022-07477); St. John’s, Botanic Garden, 23 August to 4 Sept. 1999, D. Larson (2, DLC, Accession Nos 12-09, 17-17596; latter determination confirmed using DNA Barcode - BOLD: CCDB-28535-D07); St. John’s, Bowering Park, 47.5273°N, 52.5717°W, 2 August 2022, sweep of vegetation, D. Langor (1, DLC, Accession No. 17-22049; determined by DWL); Ibid., 47.528°N, 52.749W°W, 20 August 2022 (1, DLC, Accession No. 17-22050; determined by DWL); St. John’s, Mount Scio, 12 Oct. 2002 (1, DLC, Accession No. 12-13); St. John’s, Newfoundland Drive, 47.6010°N, 52.7117°W, 83 m, 20 June 2009, sweep, D. Langor (1, CMN, DLC Accession No. 12-16; 4, DLC, Accession Nos 12-15, 17-17615 to 17-17617; determinations of three specimens confirmed using DNA Barcode - BOLD: CCDB-28535-D08 [12-15], CCDB-28535-D09 [17-17615] and CCDB-28535-D10 [17-17616]); St. John’s, Oxen Pond Botanic Garden, Sept. 2000 (1, DLC, Accession No. 17-17611; 1, NFRC, Accession No. NFRC-2022-07473); Ibid., Oct. 2000, S. Lilly (1, DLC, Accession No. 12-11); Stephenville Crossing, 48.513°N, 58.454°W, 3 m, 22 June 2010, sweep of vegetation on coastal sand dunes, David Langor (1, DLC, Accession No. 12-22); York Harbour, 49.0555°N, 58.3687°W, 2 m, 28 June 2010, sweep shoreline vegetation, David Langor (2, DLC, Accession Nos 12-21, 17-17620).

### ﻿CURCULIONIDAE

#### Ceutorhynchinae: Ceutorhynchini

***Ceutorhynchusamericanus* Buchanan, 1937**

This Nearctic species is newly recorded from NL based on one specimen collected in NF. It is otherwise widely distributed in Canada from Yukon Territory and British Columbia to Nova Scotia (Bousquet et al. 2013). Buchanan (1937) gives a list of plants, all Brassicaceae, on which adults have been collected. These are as follows: radish, horse-radish, *Lepidium* sp., cultivated mustard, mustard, and Chinese cabbage. Adults have also been reared from *Lepidiumvirginicum* L. (Buchanan 1937).

**Specimens examined**: Newfoundland: Gros Morne National Park, Shallow Bay Beach, 14 July 2005, Sand Dune Ecosystem Study, Pitfall Trap 6, semi-stable vegetation on coastal sand dunes, Shelley Pardy (1, NFRC, Accession No. NFRC-2022-07175; determined by RSA).

***Ceutorhynchusomissus* Fall, 1917**

This Nearctic species is newly recorded from NL with a single specimen from NF. It is distributed in Canada from Alberta to Nova Scotia (Bousquet et al. 2013). Host plants are in Brassicaceae.

**Specimens examined**: Newfoundland: St. John’s, 14 July 1949, W.J. Brown (1, CNC, Accession No. 04-1841; determined by PB).

***Ceutorhynchusoregonensis* Dietz, 1896**

This Nearctic species is newly recorded from NL based on a specimen from northern NF. In Canada, it is widely distributed from Yukon Territory and British Columbia to Nova Scotia (Bousquet et al. 2013), with the Maritime province records only recently reported by Majka et al. (2007b). Anderson (1997) mentions that a number of adults of this species were collected from *Rorippaislandica* (Oeder) Borbás (Cruciferae) in Utah.

**Specimens examined**: Newfoundland: St. Anthony, 51.37°N, 58.60°W, 10 July 2008, sweep sample #8, Goulet, Boudreault and Badiss (1, NFRC, Accession No. NFRC-2022-07183; determined by RSA).

#### Ceutorhynchinae: Cnemogonini

***Cnemogonuslecontei* Dietz, 1896**

This Nearctic species is newly recorded from NL based on material from LB. In Canada, it is reported from Yukon Territory and British Columbia to New Brunswick. The species may be associated with plants in the family Onagraceae (Korotyaev and Anderson 2002).

**Specimens examined**: Labrador: Goose Bay, 2 August 1982, M. Colbo (1, NFRC, Accession No. NFRC-2022-07198; determined by PB; determination confirmed by DNA barcode – BOLD: CCDB-28535-H02); Goose Bay vicinity, 53.2889°N, 60.3810°W, 5 August 2008, sweep of roadside flowers, G.R. Pohl and D.W. Langor (1, NFRC, Accession No. NFRC-2022-07182; determined by PB; determination confirmed by DNA barcode – BOLD: CCDB-28535-H01).

#### Ceutorhynchinae: Phytobiini

***Pelenomusfuliginosus* (Dietz, 1896)**

This Nearctic species is newly recorded from NL based on material from LB. In Canada, it has been reported from British Columbia to New Brunswick (Bousquet et al. 2013). Species of *Pelenomus* are generally associated with *Polygonum* (Polygonaceae).

**Specimens examined**: Labrador: Mealy Mountains, 53.67°N, 58.87°W, July 2002, Anions, Sutton and Quicke (1, DLC, Accession No. 12-221; 2, NFRC, Accession Nos NFRC-2022-07199 to 2022-07200; determined by PB).

***Rhinoncusbruchoides* (Herbst, 1784)**

This Palearctic species is distributed throughout Europe and Asia (Alonso-Zarazaga et al. 2017). In Canada, this adventive species is newly documented from NL based on a series of three BugGuide photos (#225470 to #225472) of a specimen from NF. The earliest records from North America are from 1979 in Delaware and Maryland (Hoebeke and Whitehead 1980). In Canada, it is known from Ontario, Quebec, New Brunswick, and Nova Scotia (Bousquet et al. 2013; Webster et al. 2016). Hosts include species of *Polygonum*, *Oenanthe* (Apiaceae), and *Chaerophyllum* (Apiaceae) (Hoebeke and Whitehead 1980).

**Specimens examined**: Newfoundland: Logy Bay, 47.6305°N, 52.6876°W, 15 September 2008, Mardon Erbland (1, photo posted on BugGuide, see https://bugguide.net/node/view/225470/bgpage; determined by RSA).

***Rhinoncuspericarpius* (Linnaeus, 1758)**

Formerly known by the name *Rhinoncuscastor* (Fabricius, 1792), this Palearctic species is distributed throughout Europe and Asia (Alonso-Zarazaga et al. 2017) and is adventive in North America according to Bousquet et al. (2013), although Alonso-Zarazaga et al. (2017) do not record it as such. In Canada, it was previously known from NF but is herein newly recorded from LB. It is widely distributed in North America, and in Canada it is recorded from British Columbia to NF (Bousquet et al. 2013). Recorded hosts of this species are sheep’s sorrel (*Rumexacetosella*), alfalfa (*Medicagosativa* L.; Fabaceae), and water dropworts (*Oenanthe* spp.) (Hoebeke and Whitehead 1980). The specimens previously determined as *R.pericarpius* and reported as such in Bousquet et al. (2013) were later determined to be *R.leucostigma* (Marsham, 1802).

**Specimens examined**: Labrador: Goose Bay, 5 July 1980, R. Morris (2, AAFC; determined by DWL); Goose Bay, vicinity of Mud Lake, 53.30570°N, 60.26812°W, 15 m, 29 July 2008, weedy shore of Churchill River, G.R. Pohl and D.W. Langor (1, NFRC, Accession No. NFRC-2022-07205; determined by PB as *R.castor*; Happy Valley, “Maxwells”, 53.29513°N, 60.30249°W, 15 m, MV light on building near Churchill River, at dusk, G.R. Pohl and D.W. Langor (1, NFRC, Accession No. NFRC-2022-07207; determined by PB).

#### Ceutorhynchinae: Scleropterini

***Prorutidosomadecipiens* (LeConte, 1876)**

This Nearctic species is newly recorded from NL based on material from both NF and LB. In Canada, it is widely distributed from Yukon Territory and British Columbia to Prince Edward Island (Bousquet et al. 2013). Adults have been associated with *Populus* (Salicaceae) (Anderson 1997).

**Specimens examined**: Newfoundland: Burnt Cape, 51.573°N, 55.754°W, 10–24 July 2003, pitfall trap in cow parsnip patch, Site 1-3, A.M. Hynes (1, DLC, Accession No. 12-222; 1, NFRC, Accession No. NFRC-2022-07206; determined by RSA); Burnt Cape, 51.573°N, 55.756°W, July to August 2003, pitfall trap in crowberry patch, Site 1-1, A.M. Hynes (2, DLC, Accession Nos 17-17943; 1, NFRC, Accession No. NFRC-2022-07203; determined by DWL); same except Site 2-1 (1, NFRC, Accession No. NFRC-2022-07204; determined by DWL). Labrador: Mealy Mountains, 53°38'N, 58°52'W, July 2002, Anions, Sutton and Quicke (2, DLC, Accession Nos 12-224, 17-17945; 1, NFRC, Accession No. NFRC-2022-07205; determined by PB; determinations confirmed by DNA barcodes – BOLD: CCDB-28534-C07, -C08, -C09, respectively).

#### Cossoninae: Rhyncolini

***Rhyncolusbrunneus* Mannerheim, 1843**

This Nearctic species was previously known from NF but herein is recorded from LB for the first time. In Canada, it is distributed from Yukon Territory and British Columbia to Prince Edward Island (Bousquet et al. 2013). Adults are found under the bark of dead Pinaceae (Anderson 1997).

**Specimens examined**: Labrador: Ossak Camp, 53.4233°N, 65.0129°W, 1 August 2004, pitfall trap, S. Pardy and R. Perry (1, DLC, Accession No. 12-234; determined by DWL).

***Rhyncolusmacrops* Buchanan, 1946**

This Nearctic species is recorded from NL for the first time based on a specimen from NF. It was previously reported in Canada from British Columbia to Prince Edward Island (Bousquet et al. 2013). It feeds in the phloem of various conifers, especially *Abies* and *Pinus* (Pinaceae) (Buchanan 1946).

**Specimens examined**: Newfoundland: Notre Dame Provincial Park, 49.116°N, 55.079°W, 30 July 2011, pitfall trap, conifer forest, L. Pollett (1, NFRC, Accession No. NFRC-2022-07227; determined by RSA).

#### Curculioninae: Acalyptini

***Acalyptuscarpini* (Fabricius, 1792)**

This species is known in Canada from Yukon Territory and British Columbia to Nova Scotia (Bousquet et al. 2013). Here we record this species for the first time from the province based on material from LB. Adults are associated with species of *Salix* (Salicaceae), the larvae developing in the catkins (Anderson 1997).

**Specimens examined**: Labrador: Goose River, 7 km north of Goose Bay, 53.39229°N, 60.42094°W, 20 m asl, 30 July 2008, sweep of *Salix* on sand flats, D. Langor and G. Pohl (2, DLC, Accession Nos 12-50, 17-17693; 3, NFRC, Accession Nos NFRC-2022-07120 to 2022-07122 determined by DWL).

**Curculioninae: Anthonomini**

**Anthonomus (Anthonomus) lecontei Burke, 1975**

This Nearctic species is reported for the first time from NL with a single specimen from NF. It is widely distributed in Canada from British Columbia to Prince Edward Island (Bousquet et al. 2013). Burke (1968) described pupae of *A.lecontei* (as *Anthonomusscutellatus* Gyllenhal) from flower heads of *Aster* sp. (Asteraceae), probably *Asterdivaricatus* (Nutt.) Kuntze, from Silver Springs, Maryland. Ahmad and Burke (1972) described the larva of the species from the same plant at the same locality.

**Specimens examined**: Newfoundland: Portugal Cove, 47.6206°N, 52.8365°W, 15 m asl, 20 July 2006, sweep of vegetation, D. Langor (1, NFRC, Accession No. NFRC-2022-07126; determined by RSA and confirmed using DNA barcode – BOLD: CCDB-28535-E04).

**Anthonomus (Paranthonomus) rubidus LeConte, 1876**

This Nearctic species is newly recorded from the province based on material from NF. It is recorded from British Columbia, Ontario, and Quebec in Canada (Bousquet et al. 2013). Adults have been recorded from various Betulaceae, Fagaceae, Juglandaceae and Rosaceae (Clark 1987).

**Specimens examined**: Newfoundland: South Branch, 17 July 1982, A. Raske (2, NFRC, Accession Nos NFRC-2022-07128 and 2022-07129; determined by RSA).

**Anthonomus (Tachypterellus) quadrigibbus Say, 1832**

This Nearctic species is newly recorded from the province based on specimens from NF. In Canada, it is recorded from British Columbia to Nova Scotia (Bousquet et al. 2013). This species develops in fruits of various Rosaceae (Burke and Anderson 1989). In NF, specimens were collected from the fruit of pin cherry, *Prunuspensylvanica* L.f.

**Specimens examined**: Newfoundland: St. John’s, Bowering Park, 47.525°N, 52.749°W, 30 June 2012, sweep sample, D. Langor and G. Pohl (1, NFRC, Accession No. NFRC-2022-07127; determined by RSA); Ibid. 47.5273°N, 52.7517°W, 2 August 2022, D. Langor [4, DLC, Accession Nos 17-22008 to 17-22011; 1, NFRC, Accession No. NFRC-2022-07482; determined by DWL); Ibid. 47.528°N, 52.749W, 20 July 2019, D. Langor (1, NFRC, Accession No. NFRC-2022-07483); Ibid. site 2, 47.5278°N, 52.7480W, 17 August 2022, pin cherry fruit, D. Langor (4, DLC, Accession Nos 17-22012 to 17-22015; 1, NFRC, Accession No. NFRC-2022-07483; determined by DWL); St. John’s, Waterford Valley High School, 47.531°N, 52.753°W, 20 July 2019, sweep sample, D. Langor (1, NFRC, Accession No. NFRC-2022-07484; determined by DWL).

#### Curculioninae: Ellescini

***Dorytomuslaticollis* LeConte, 1876**

The first NL record of this Nearctic species is herein reported from LB. This species is distributed from British Columbia to Nova Scotia (Bousquet et al. 2013) and feeds on trembling aspen, *Populustremuloides* Michx. (O’Brien 1970).

**Specimens examined**: Labrador: 35 km west of Goose Bay, 53.2687°N, 60.8626°W, 50 m asl, 2 July 2009, attracted to MV light, mixed forest, D. Langor and D. Macaulay (1, NFRC, Accession No. NFRC-2022-07143; determined by RSA and confirmed by DNA barcode – BOLD: CCDB-28535-E06).

***Dorytomusparvicollis* Casey, 1892**

This Nearctic species is herein reported for the first time from NL based on a single specimen from LB. In Canada, the species is recorded from British Columbia to Nova Scotia (Bousquet et al. 2013). Trembling aspen is the only known host of this species (O’Brien 1970).

**Specimens examined**: Newfoundland: Glover’s Harbour, 1 August 1968, FIS 68-1-0772(01), *Populustremuloides* (1, NFRC, Accession No. NFRC-2022-07144; determined by RSA; DNA barcode was inconclusive – BOLD: CCDB-28535-E06).

***Dorytomusrufulus* (Mannerheim, 1853)**

This Nearctic species is reported for the first time from NL based on a single specimen from LB. The species is distributed in Canada from Yukon Territory and British Columbia to Nova Scotia (Bousquet et al. 2013). Willows (*Salix* spp.) are the hosts of this species (O’Brien 1970).

**Specimens examined**: Labrador: Goose Bay, 24–26 Sept. 1981, M. Colbo (1, NFRC, Accession No. NFRC-2022-07142; determined by RSA and confirmed by DNA barcode – BOLD: CCDB-28535-E07).

***Dorytomusvagenotatus* Casey, 1892**

Although previously reported from NF (Bousquet et al. 2013), this Nearctic species is herein reported for the first time from LB. It is recorded in Canada from Yukon Territory and British Columbia to NF (Bousquet et al. 2013). The known hosts of this species are *Populustremuloides* and *Populusgrandidentata* Michx. (bigtooth aspen) (O’Brien 1970).

**Specimens examined**: Labrador: Goose Bay, shore of Churchill River, 53.29683°N, 60.28221°W, 10 m asl, 29 July 2008, sandy river shore, G. Pohl and D. Langor (1, DLC, Accession No. NFRC-2022-07146; determined by DWL and confirmed by DNA barcode – BOLD: CCDB-28535-E09); Happy Valley, Maxwell’s Restaurant, 53.29513°N, 60.30249°W, 15 m asl, 29 July 2008, collected at MV light at dusk, G. Pohl and D. Langor (1, NFRC, Accession No. NFRC-2022-07147; determined by RSA and confirmed by DNA barcode – BOLD: CCDB-28535-E08).

#### Curculioninae: Mecinini

***Mecinuspascuorum* (Gyllenhal, 1813)**

This Palearctic weevil is native to the Palearctic where it is widely distributed, and it has been introduced to Africa, North America, and Australia (Alonso-Zarazaga et al. 2017). In Canada, it has been recorded from British Columbia and from Ontario to Nova Scotia. Here we record it from NL for the first time based on one specimen collected in western NF. This species feeds on *Plantagolanceolata* L. (Plantaginaceae) (Anderson et al. 2018).

**Specimens examined**: Newfoundland: South of Frenchman’s Cove on Route 450, 49.0499°N, 58.1552°W, 8 August 2022, sweep of vegetation along disturbed roadside, D. Langor (1, NFRC, Accession No. NFRC-2022-07486; determined by RSA).

#### Curculioninae: Rhamphini

***Orchestespallicornis* Say, 1832**

This Nearctic species is recorded from British Columbia to NF (Bousquet et al. 2013). Although originally recorded from NF by O’Brien and Wibmer (1982), Anderson (1989) asserted that he had not seen specimens substantiating this record, nor have we seen specimens or verified locality records of this species from NF. We hereby report the first record of this species from LB, which also represents the first published locality record for the entire province. Larvae feed on Rosaceae, including *Crataegus*, *Prunus*, *Pyrus* and likely *Amelanchier* (Anderson 1989).

**Specimens examined**: Labrador: Goose Bay, military base, 53.29833°N, 60.43562°W, 15 m, 28 July 2008, weedy Salix/grass clearing, Greg Pohl & David Langor (1, NFRC, Accession No. NFRC-2022-07160; determined by RSA).

#### Curculioninae: Styphlini

***Orthochaetessetiger* ([Beck], 1817)**

**Figure 1.**
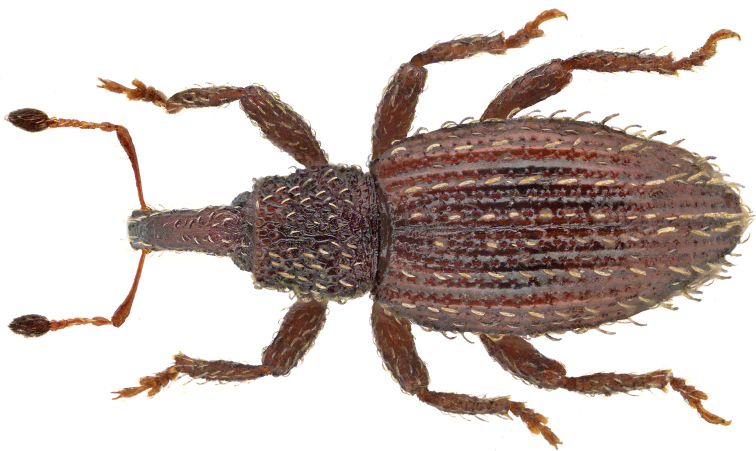
Dorsal habitus photo for *Orthochaetessetiger* ([Beck]). Body length is 2.5 mm. [photo credit: Udo Schmidt; for further details see Acknowledgements].

This Palearctic species is native to Europe (Alonso-Zarazaga et al. 2017) and is herein reported for the first time as adventive in Canada based on specimens from NF (Fig. 1) and British Columbia. These are also the first records of the species from North America. In Britain, larvae are known to mine the leaves of many species in the families Amaryllidaceae, Asteraceae, Boraginaceae, Lamiaceae, Plantaginaceae, and Primulaceae (Pitkin et al. 2019). In the northern part of its European range, the species reproduces parthenogenetically (Ellis 2019). In Europe, adults have been located throughout the year under moss on old coniferous stumps, among rotten detritus in forest undergrowth, and in ant nests, e.g., *Lasius*, *Formica* (Formicidae) (González 1967). Adults are 2.3 - 2.9 mm long.

There are no other members of the Palearctic tribe Styphlini in North America. Adults of *O.setiger* will key out to couplet 26 (as Cyclominae) in the key to Nearctic subfamilies of Curculionidae for the USA and Canada (Anderson 2002). The genus *Orthochaetes* can be separated from Cyclominae by the combination of less well-developed postocular lobes, the lack of a metaepisternal suture, a proportionally longer elytra as compared to the length of the pronotum and the presence of erect, spatulate seta-like scales on the elytral intervals. In the key to Nearctic genera of Curculioninae, *Orthochaetes* will key to couplet 9 (as *Pachytychius*) but it lacks a tooth on the hind femur (present in *Pachytychius*).

**Specimens examined (all determined by RSA)**: British Columbia: Metchosin, summit of Camas Hill, 48°23'57"N, 123°35'44"W, 14 Dec. 1999 – 3 Jan. 2000, D. Blades, L. Rosenblood, C. Reznechenko, CH99 – 16P (1, RBCM, Accession No. ENT017-007401). Newfoundland: St. John’s, 4 Oct. 1995 (1, DLC, Accession No. 12-670); St. John’s, Botanic Garden, 23 Aug. – 4 Sep. 1999, D. Larson (1, CMN).

#### Curculioninae: Tychiini

***Tychiuspicirostris* (Fabricius, 1787)**

This Palearctic species is native to Europe and western Asia and is adventive in North America (Alonso-Zarazaga et al. 2017). In Canada, it has been reported from British Columbia to NF (Bousquet et al. 2013). Herein we provide the first report from LB. In North America and Europe, Fabaceae host plants are *Trifoliumrepens* L. (white clover) and *T.hybridum* L. (alsike clover) (Anderson and Howden 1994).

**Specimens examined**: Labrador: Goose Bay, air base, 53.301°N, 60.423°W, 46 m asl, 30 June 2009, sweep of shrubs and forbs, D. Langor (2, DLC; Accession Nos 17-17887, 17-17888; 1, NFRC, Accession No. NFRC-2022-07167; determined by DWL); Goose Bay, military base, 53.29833°N, 60.43562°W, 28 July 2008, 15 m asl, sweep of weeds, *Salix* and grasses, G. Pohl and D. Langor (6, DLC, Accession Nos 17-17879 to 17-17883, 12-160; 2, NFRC, Accession Nos NFRC-2022-07165 and 2022-07166; determined by RSA); Happy Valley, Maxwell’s Restaurant, 53.29513°N, 60.30249°W, 15 m asl, 29 July 2008, MV light, G. Pohl and D. Langor (1, DLC, Accession No. 12-153; determined by DWL).

***Tychiusstephensi* Schönherr, 1836**

This Palearctic species is native to Europe and western Asia and is adventive in North America (Alonso-Zarazaga et al. 2017). In Canada, we report it from NL for the first time based on material from NF. In Canada, it is recorded from British Columbia to Prince Edward Island (Bousquet et al. 2013). In North America this species is found on *Trifoliumpratense* L. (red clover); larvae feed on the developing seeds (Anderson and Howden 1994).

**Specimens examined**: Newfoundland: Corner Brook, mid July 2004, pine, Erika Burke (4, DLC, Accession Nos 12-158, 17-17891 to 17-17893; determined by DWL); Corner Brook, 48°57.355'N, 57°54.681'W, 12 July 2008, fallow field, sweep sample #11, Goulet, Boudreault & Badiss (4, DLC, Accession Nos 17-17920 to 17-17922, 12-169; determined by DWL and confirmed by DNA barcode – BOLD: CCDB-28535-F11 [AN: 12-169]) Curling Brook, 48.9593°N, 57.9916°W, 11 m asl, 29 June 2010, sweep of cow parsnip flowers, David Langor (1, NFRC, Accession No. NFRC-2022-07171; determined by DWL); Deer Lake, 49°11.711'N, 57°54.681'W, 12 July 2008, fallow field, sweep sample #5, Goulet, Boudreault & Badiss (12, DLC, Accession Nos 17-17902 to 17-17910, 17-17912, 12-164; 1, NFRC, Accession No. NFRC-2022-07174; determined by DWL); Gros Morne National Park, Stanleyville, 49.468°N, 57.773°W, 10 m asl, 6 July 2006, on beach near cliffs, D. Langor (6, DLC, Accession Nos 17-17896 to 17-17900, 12-162; determined by DWL); Lomond, 49.4619°N, 57.7610°W, 3 m asl, 6 July 2006, sweep sample, D. Langor (2, DLC, Accession Nos 17-17894, 17-17895; 1, NFRC, Accession No. NFRC-2022-07172; determined by DWL); Paddy’s Pond, 7 Oct. 1995 (1, DLC, Accession No. 12-156; determined by DWL); Portugal Cove, 47.6206°N, 52.8365°W, 15 m asl, 20 July 2006, sweep sample, D. Langor (1, DLC, Accession No. 12-168; determined by DWL and confirmed by DNA barcode – BOLD: CCDB-28535-F12); St. John’s, 47.598°N, 52.713°W, 18-22 July 2006, sweep of shrubs and forbs, D. Langor (1, DLC, Accession No. 17-17891; 2, NFRC, Accession Nos NFRC-2022-07169, 2022-07170; determined by DWL); St. John’s, Long Pond, 4 July 2000, N. O’Dea (2, DLC, Accession Nos 17-17890, 12-157; determined by DWL); St. John’s, Newfoundland Drive, 47.6010°N, 52.712°W, 83 m asl, 2 July 2010, sweep of vegetation, D. Langor (1, DLC, Accession No. 12-166; determined by DWL and confirmed by DNA barcode – BOLD: CCDB-28535-G03); St. John’s, Quidi Vidi Lake outlet, 47.5841°N, 52.6799°W, 15 m asl, 20 June 2009, sweep of vegetation, D. Langor (1, NFRC, Accession No. NFRC-2022-07173; determined by DWL); Stephenville Crossing, 48.513°N, 58.454°W, 3 m asl, 22 June 2010, sweep of vegetation on coastal sand dunes, D. Langor (2, DLC, Accession Nos 17-17910, 12-167; determined by DWL and confirmed by DNA barcodes – BOLD: CCDB-28535-G01 and CCDB-28535-G02, respectively).

#### Dryophthorinae: Rhynchophorini

***Sitophiluszeamais* Motschulsky, 1855**

This cosmopolitan species is adventive in North America and has been recorded from Manitoba to Quebec (Bousquet et al. 2013).

**Specimens examined**: Newfoundland: St. John’s, 4 November 1985, Ray Morris (1, CNC, Accession No. CNC COLEO 00116857; determined by DNA barcoding – CNC774-11).

#### Entiminae: Phyllobiini

***Phyllobiusoblongus* (Linnaeus, 1758)**

This Palearctic species, commonly called the European snout weevil, is native to Europe and western Asia, and is adventive in North America (Alonso-Zarazaga et al. 2017). We report it herein for the first time from NL, based on specimens from NF. It has been recorded in Canada from British Columbia in the west and from Ontario to Prince Edward Island in the east (Bousquet et al. 2013). Adults feed on the leaves of a wide variety of shade and fruit trees in the families Betulaceae (*Betula*), Rosaceae (*Malus*, *Prunus*, *Pyrus*), Salicaceae (*Populus*, *Salix*), Sapindaceae (*Acer*), and Ulmaceae (*Ulmus*), and larvae feed on the roots of various plants (Bright and Bouchard 2008, and references therein).

**Specimens examined (all determined by DWL)**: Newfoundland: Corner Brook, near Prince Edward Park, 48.967°N, 57.889°W, 2 m asl, 29 June 2010, sweep of vegetation, D. Langor (1, DLC, Accession No. 12-485); Marble Mountain, 48.9492°N, 57.8302°W, 12 m asl, 19 June 2010, UV light in mixed forest, D. Langor & D. Macaulay (1, NFRC, Accession No. NFRC-2022-07288); Pasadena, 49.0121°N, 57.6106°W, 2 m asl, 25 June 2011, gravel and sand on river bank, D. Langor & G. Pohl (1, DLC, Accession Nos 17-18297; 1, NFRC, Accession No. NFRC-2022-07289).

#### Entiminae: Polydrusini

***Pachyrhinuselegans* (Couper, 1865)**

This Nearctic species is distributed in Canada from British Columbia to Nova Scotia (Bousquet et al. 2013). We report it for the first time from the province of NL based on specimens from NF. This species occurs mainly on eastern white pine, *Pinusstrobus* L.a., which occurs in NF, and occasionally feeds on other pine species elsewhere in Canada (Bright and Bouchard 2008).

**Specimens examined (all determined by DWL)**: Newfoundland: Pasadena, east of on TCH, 49.0706°N, 57.5592°W, 5 August 2022, sweep of vegetation, D. Langor (2, DLC, Accession Nos 17-22080 and 17-22081); Upper Ferry, near bridge, 47.8496°N, 59.2477°W, 10 August 2022, sweep of vegetation at roadside, D. Langor (1, NFRC, Accession No. NFRC-2022-07487).

***Polydrususcervinus* (Linnaeus, 1758)**

This Palearctic species is native to Europe and western Siberia and is adventive in North America (Alonso-Zarazaga et al. 2017). Here we record it for the first time from NL based on specimens from NF. In Canada, the species is recorded from Ontario, Nova Scotia, and Prince Edward Island (Bousquet et al. 2013). Adults feed on the leaves of *Acer*, *Alnus*, *Betula*, *Corylus*, *Malus*, *Prunus*, *Populus*, *Quercus*, and *Salix*; the hosts of larvae are not fully documented but include roots of orchard grass, *Dactylisglomerata* L. (Bright and Bouchard 2008, and references therein).

**Specimens examined**: Newfoundland: Tompkins (trail near), 47.781°N, 59.231°W, 20 m asl, 23 June 2010, sweep sample, D. Langor (3, DLC, Accession Nos 17-18326, 17-18327, 12-492; 2, NFRC, Accession Nos NFRC-2022-07297 and 2022-07298; determined by DWL).

***Polydrususformosus* (Mayer, 1779)**

This Palearctic species is native to Europe and is adventive in North America (Alonso-Zarazaga et al. 2017). Herein we record the species for the first time from NL based on specimens from NF where it is very widespread and often abundant. It is recorded in Canada from British Columbia in the west and Ontario to Prince Edward Island in the east (Bousquet et al. 2013). Adults of this species have been collected in canola fields and on wild radish in pea fields (Pinski et al. 2005). Much information about this species is published under the name *Polydrusussericeus* (Schaller), now a junior synonym.

**Specimens examined (determined by DWL unless otherwise noted)**: Newfoundland: Blow Me Down, 49.061°N, 58.232°W, 6 July 2012, sweep of plants around river trailhead, J. Heron (2, DLC, Accession Nos 17-18319, 17-18320); Blue Pond Park, 48.783°N, 58.087°W, 212 m asl, 25 June 2010, sweep of vegetation along lake shore, D. Langor (3, DLC, Accession Nos 17-18313 to 17-18315; 2, NFRC, Accession Nos NFRC-2022-07292 and 2022-07295); Cheeseman Provincial Park, 47.628°N, 59.273°W, 8 July 2012, D. Langor and G. Pohl (1, DLC, Accession No. 17-18322; determination confirmed by DNA barcode – BOLD: CCDB-28536-D03); Cormack, 49.2679°N, 57.4408°W, 12 August 2022, sweep of vegetation along disturbed roadside, D. Langor (1, DLC, Accession No. 17-21990); Corner Brook, 48.975°N, 57.9°W, 10 July 2008, sweep of fallow field, Goulet, Boudreault, and Badiss (3, DLC, Accession Nos 17-18310, 17-18311, 12-490); Corner Brook, 49.9453°N, 57.9168°W, 13 July 2006, sweep sample, D. Langor (8, DLC, Accession Nos 17-18303 to 17-18309, 12-488; 1, NFRC, Accession No. NFRC-2022-07291); Corner Brook, 24 July 2002, sweep of maples, R. Feng (1, DLC, Accession No. 17-18317; 1, NFRC, Accession No. NFRC-2022-07293); Corner Brook, 24 July 2002, sweep of white birch, R. Feng (1, DLC, Accession No. 17-18316); Gander, near Silent Witness Memorial, 48.9177°N, 54.5688°W, 4 August 2022, sweep of vegetation in disturbed lot, D. Langor (3, DLC, Accession Nos 17-21996 to 17-21998); George’s Lake, 48.780°N, 58.101°W, 134 m asl, 9 July 2012, brook margin, D. Langor and G. Pohl (1, DLC, Accession No. 17-18321; determination confirmed by DNA barcode – BOLD: CCDB-28536-D04); Grand Falls, Sanger Park, 48.9258°N, 55.6407°W, 5 August 2022, sweep of vegetation along forest trail (semi-natural), D. Langor (2, DLC, Accession Nos 17-21999 and 17-22000); Gros Morne National Park, James Callaghan Trail, 49.5686°N, 57.8302°W, 39 m, 16 July 2013, forest, malaise trap, Anderson (1, CBG, determination by DNA barcode – BOLD: BIOUG09982-H03); Ibid. 27 August 2013, R. Reid (1, CBG, determination by DNA barcode – BOLD: BIOUG10587-A01); Marble Mountain near Humber River, 48.952°N, 57.836°W, 24 m asl, 24 June 2010, at MV light, D. Langor (1, DLC, Accession No. 12-489); Port aux Basques, 47.5841°N, 59.1406°W, 9 August 2022, sweep of vegetation in field and forest edge, D. Langor (3, DLC, Accession Nos 17-21985 to 17-21987); Port Saunders, Route 430, 50.6496°N, 57.2795W, 6 August 2022, sweep of vegetation in disturbed open area, D. Langor (1, DLC, Accession No. 17-21983); Rocky Harbour, 49.5848°N, 57.9066°W, 6 August 2022, sweep of vegetation on disturbed land, D. Langor (3, DLC, Accession Nos 17-22001 to 17-22003); Searston, 47.8299°N, 59.3064°W, 10 August 2022, sweep of vegetation on disturbed roadside and field, D. Langor (1, DLC Accession No. 17-21984); Steady Brook, 48.9487°N, 57.8304°W, 20 m asl, 13 August 2008, sweep sample and pond edge, D. Langor (4, DLC, Accession Nos 17-18299, 17-18300, 17-18302, 12-487; 1, NFRC, Accession No. NFRC-2022-07290; determined by RSA); Terra Nova National Park, visitors center, 48.5796°N, 53.9471W, 4 August 2022, sweep at vegetation at forest edge, D. Langor (3, DLC, Accession Nos 17-22004 to 17-22006); Upper Harbour near Deer Lake, 49.193°N, 57.434°W, 9 July 2012, sweep of shoreline vegetation, D. Langor and G. Pohl (1, NFRC, Accession No. NFRC-2022-07294); Wreckhouse, 47.7091°N, 59.3073°W, 9 August 2022, sweep of vegetation on disturbed roadside and in natural meadow, D. Langor (1, DLC, Accession No. 17-21988); York Harbour, 49.056°N, 58.369°W, 10 m asl, 6 July 2012, D. Langor (2, DLC, Accession Nos 17-18323 and 17-18324; determination confirmed by DNA barcodes – BOLD: CCDB-28536-D02, CCDB-28536-D01, respectively).

#### Entiminae: Sitonini

***Sitonacylindricollis* Fåhraeus, 1840**

The natural range of this Palearctic species is Europe, western Asia, and North Africa (Alonso-Zarazaga et al. 2017). Commonly known as the sweet-clover weevil, it was first found in North America (Hemmingford, QC) in 1924 (Bright and Bouchard 2008). It is now found throughout the USA, and in Canada it is recorded from Yukon Territory and British Columbia to Prince Edward Island (Bright 1994; Bousquet et al. 2013). Here we report it for the first time from NL based on material from NF. The main host of this weevil is sweet clover (*Melilotus* spp.), but it can also be occasionally found on *Medicagosativa*, *Trifoliumhybridum* and *Medicagolupulina* L. (black medick) (Bright 1994).

**Specimens examined**: Newfoundland: Corner Brook, 48.975°N, 57.9°W, 10 July 2008, fallow field, sweep sample #11, Goulet, Boudreault & Badiss (2, DLC, Accession Nos 17-18416 and 12-611; 1, NFRC, Accession No. NFRC-2022-07250; determined by DWL); St. John’s, 29 September 1980 (1, DLC, Accession No. 17-17580; determined by RSA); St. John’s, 15 August 1997, P. Dixon (2, AAFC; determined by DWL); St. John’s, 12 July 1999, J. Coombs (1, AAFC; determined by DWL); Ibid., 20 August 1999 (1, AAFC; determined by DWL).

**Lixinae: Cleonini**

***Stephanocleonusplumbeus* LeConte, 1876**

This Nearctic species was reported from NF by Anderson (1987) and is now recorded from LB for the first time. In Canada, it is distributed from Alberta to NF (Anderson 1987; Bousquet et al. 2013). There is no recorded information about natural history.

**Specimens examined**: Labrador: Happy Valley – Goose Bay, 6 June 2005, S. Pardy-Moores (1, DLC, Accession No. 12-666; determined by DWL and confirmed by DNA barcode – BOLD: CCDB-28536-E09).

#### Mesoptiliinae: Magdalidini

***Magdalisalutacea* LeConte, 1878**

This Nearctic species is reported for the first time from the province based on specimens from both NF and LB. It is recorded in Canada from Yukon Territory and British Columbia to New Brunswick (Bousquet et al. 2013). This weevil has been reared from *Piceaengelmanni* Parry ex Engelm. (Pinaceae) in Colorado (Anderson 1997).

**Specimens examined (all determined by RSA)**: Newfoundland: Gander, 30 June 1949, W. J. Brown (1, CNC). Labrador: Goose Bay, 10 August 1948, W. E. Beckel (1, CNC).

***Magdalisgentilis* LeConte, 1876**

This Nearctic species is reported from the province for the first time based on specimens from NF. In Canada, it is recorded from Yukon Territory and British Columbia to Nova Scotia (Bousquet et al. 2013). Adults have been reported from various species of *Pinus* and *Picea* (Pinaceae) (Anderson 1997).

**Specimens examined**: Newfoundland: Birchy Lake (south side), 28 June 1972, *Pinusstrobus*, FIS 72-1-0098 (1, CFS; determined by RSA).

#### Molytinae: Hylobiini

***Hylobiuswarreni* Wood, 1957**

This species was originally reported from NF in 1991 (McNamara 1991a) but was removed from the 2013 checklist (Bousquet et al. 2013) as verified specimens had not been seen. However, this Nearctic species is hereby reinstated as present in both NF and LB. It is recorded in Canada from British Columbia to Nova Scotia (Bousquet et al. 2013). The species is associated with conifers, primarily *Pinus* and *Picea* (Cerezke 1994).

**Specimens examined (determined by DWL except where noted)**: Newfoundland: Bonavista, 26 August 1960, FIS 60-1740(01), ex. Scots pine (1, CFS); Butterpot Provincial Park, 47.381°N, 53.044°W, 4 August 2012, forest, pitfall trap #2, A. Pretty (1, NFRC, Accession No. NFRC-2022-07363); Cormack, 12 June 1979, Lot 2, Larson and Swales (1, DLC, Accession No. 12-686); Gales Brook, 22 June 1977 (1, DLC, Accession No. 12-675); Gander, July 1979 (3, DLC, Accession Nos 12-681, 17-20374, 17-20375); Grand Falls (near), 48.974°N. 55.569°W, 44 m asl, 1-10 July 2012, pitfall trap in mixed wood forest, D. Langor and G. Pohl (1, NFRC, Accession No. NFRC-2022-07361; determined by RSA); Gros Morne National Park, Bakers Brook, July-August 2000, pitfall trap in tuckamoor, N. Chalk (1, DLC, Accession No. 12-692; identity confirmed by DNA Barcode – BOLD: CCDB-28536-F02); Lockston Path Provincial Park, 48.430°N, 53.361°W, 26 August 2011, pitfall trap in conifer forest, P. Perry (1, DLC, Accession No. 17-20379); Notre Dame Provincial Park, 49.116°N, 55.079°W, 13 September 2011, pitfall trap in conifer forest, L. Pollett (1, DLC, Accession No. 17-20377); Ibid., 2 July 2011 (1, NFRC, Accession No. NFRC-2022-07362); Portugal Cove, Indian Meal Line, 26 June 1981, D. Larson (1, DLC, Accession No. 12-690); Portugal Cove, Indian Meal Line, 27 May 1979, D. Larson (1, DLC, Accession No. 12-689); Terra Nova National Park, Wings Brook, 17 July 1982 (1, DLC, Accession No. 12-694). Labrador: Churchill River, 53.176°N, 60.948°W, 1 July 2009, shoreline of river, D. Langor and S. Pardy-Moores (1, DLC, Accession No. 12-691); Mealy Mountains, 15 July 2005, SWP PF6PF2 (1, NFRC, Accession No. NFRC-2022-07359); Middle Brook, 53.3785°N, 63.1429°W, 4-18 July 2005, pitfall trap in boreal forest, S. Pardy (1, DLC, Accession No. 12-698); Muskrat Falls, 53.2606°N, 60.7844°W, 5-19 July 2005, pitfall trap in boreal forest, S. Pardy (1, NFRC, Accession No. NFRC-2022-07360); Ibid, 17 June – 5 July 2005 (1, DLC, Accession No. 17-20376); St. Lewis, 52.3961°N, 55.7057°W, 5-22 July 2004, pitfall trap in coastal barrens, S. Pardy (1, DLC, Accession No. 12-699).

#### Molytinae: Lepyrini

***Lepyruspalustris* (Scopoli, 1763)**

This Holarctic species is native to Europe, throughout Siberia and in northern North America (Alonso-Zarazaga et al. 2017). It was recorded from LB by McNamara (1991a); however, we have not seen the locality data or specimens on which this record is based. Subsequently, two specimens were collected from Shuldham Island. Here we report it for the first time from NF and provide a hitherto unpublished record from LB. In Canada, it is recorded from Northwest Territories and British Columbia to LB (Bousquet et al. 2013). Hoffmann (1954) notes that larvae of *L.palustris* in Europe feed on the primary roots of *Rumexobtusifolius* L. (Polygonaceae) even though adults are found frequently on various species of Salicaceae.

**Specimens examined (all determined by RSA)**: Newfoundland: Burnt Cape, 51.571°N, 55.756°W, 10-24 July 2003, pitfall trap in crowberry lawn, Site 1-1, A.M. Hynes (1, NFRC, Accession No. 12-702; 3, NFRC, Accession Nos NFRC-2022-07366 to 2022-07368); Ibid, *Dryas* rock garden, Site 1-4 (1, DLC, 17-20387). Labrador: Shuldham Island, 28-29 July 2006, Michael Burzynski (1, DLC, Accession No. 17-20388; 1, NFRC, Accession No. NFRC-2022-07369).

#### Molytinae: Pissodini

***Pissodesrotundatus* LeConte, 1876**

This Nearctic species has a transcontinental distribution and was previously recorded from NF (Bousquet et al. 2013). Herein we report it for the first time from LB, and its presence there is unsurprising. It was previously recorded in Canada from Yukon Territory and British Columbia to NF (Bousquet et al. 2013). Larvae breed in the phloem and cambium of the boles of recently dead or weakened *Picea* and *Pinus* (Hopkins 1911).

**Specimens examined**: Labrador: Churchill Road, 14 July 1976, FIS 76-1-0564, ex. *Piceamariana*, W. Sutton (1, CFS; determined by DWL).

***Pissodesstriatulus* (Fabricius, 1775)**

This transcontinental Nearctic species is already known from NF where it is widely distributed, but herein we report it for the first time from LB. In Canada, it is distributed from British Columbia to NF (Bousquet et al. 2013). Larvae breed in the phloem and cambium of *Abies* (Pinaceae) (Hopkins 1911).

**Specimens examined**: Labrador: 0.25 mi N of Goose River Bridge, 13 July 1977, FIS 77-1-0490-02, ex *Abiesbalsamea*, K.E. Pardy (1, CFS; determined by DWL); km 34 of Grand Lake Road near Goose Bay, 24 July 1986, K.E. Pardy (1, NFRC, Accession No. NFRC-2022-07387; determined by DWL and confirmed by DNA barcode – BOLD: CCDB-28536-G04); Minipi Lake, 1 July 1965, FIS 65-0324-01, ex *Abiesbalsamea* (1, CFS; determined by DWL); Muskrat Is., 15 km W. Goose Bay, 5 August 1984, ex. *Abiesbalsamea*, A.G. Raske (3, CFS; determined by DWL; two specimens confirmed by DNA barcodes – BOLD: CCDB-28536-G01, -G02).

#### Scolytinae: Dryocoetini

***Dryocoetesaffaber* (Mannerheim, 1852)**

This Nearctic species is known from NF but herein we report it for the first time from LB. In Canada, it is recorded from Yukon Territory and British Columbia to NF (Bousquet et al. 2013). It breeds in the phloem of various conifers, mainly *Picea* and *Pinus* but also *Larix* (Pinaceae) and *Abies* (Bright 1976).

**Specimens examined (all determined by DWL)**: Labrador: Cartwright Highway, 52.9187°N, 60.2360°W, 5 August 2008, on log deck (5, DLC, Accession Nos 17-20468 to 17-20472; 2, NFRC, Accession Nos NFRC-2022-07408 and NFRC-2022-07409; determination confirmed by DNA barcode – BOLD: CCDB-28537-A09 [A.N. 17-20470], CCDB-28537-A10 [A.N. 17-20471]); Cartwright Highway, 53.0742°N, 60.5082°W, 5 August 2008, ex spruce logs under bark (7, DLC, Accession Nos 17-20473 to 17-20479; 1, NFRC, Accession No. NFRC-2022-07410; determination confirmed by DNA barcode – BOLD: CCDB-28537-A11 [A.N. 17-20478]); ca. 35 km W Goose Bay, 53.2687°N, 60.8626°W, 50 m asl, 2 July 2009, MV light in mixed forest, D. Langor and D. Macaulay (1, DLC, Accession No. 17-20485), Labrador City ski hill, 18 July 1981, Lot 1, M. Colbo and D. Larson (2, DLC, Accession Nos 12-1685 and 17-20465); Muskrat Falls, 53.254°N, 60.777°W, 114 m asl, 30 June 2009, MV light, D. Langor and D. Macaulay (4, DLC, Accession Nos 17-20481 to 17-20484).

#### Scolytinae: Hylastini

***Scierusannectans* LeConte, 1876**

This Nearctic species was previously recorded from NF and is here reported from LB for the first time. The species is recorded in Canada from Northwest Territories and British Columbia to NF by Bousquet et al. (2013). It breeds in the phloem of *Picea*, *Abieslasiocarpa* (Hook.) Nutt. and *Pinuscontorta* Dougl. ex Loudon (Bright 1976).

**Specimens examined**: Labrador: Cartwright Highway, 53.0742°N, 60.5082°W, 5 August 2008, ex spruce logs under bark, G. Pohl and D. Langor (1, DLC, Accession No. 12-1731; determined by DWL).

**Scolytinae: Hylurgini**

***Dendroctonusrufipennis* (Kirby, 1837)**

This Nearctic species, commonly known as the spruce beetle, has a very wide distribution in North America. It is found throughout NF where it has caused outbreaks in white spruce stands, particularly along the Humber Valley (D. Langor, unpublished data). Herein we report it for the first time from LB. Larvae breed in the phloem and cambium of *Picea* spp. (Bright 1976).

**Specimens examined (all determined by DWL)**: Labrador: Cartwright Highway, 52.9187°N, 60.2360°W, 5 August 2008, on log deck, G. Pohl and D. Langor (2, DLC, Accession Nos 12-1748 and 17-20668); Charlottetown, 52.772°N, 56.115°W, 24 m asl, 26 June 2009, MV light in spruce forest, D. Langor (1, NFRC, Accession No. NFRC-2022-07423); Churchill River, 53.176°N, 60.948°W, 1 July 2009, shore of river, D. Langor and S. Pardy-Moores (1, DLC, Accession No. 12-1755).

#### Scolytinae: Ipini

***Ipsborealis* Swaine, 1911**

This Nearctic species has been recorded from NF but here we report it for the first time from LB. In Canada, it is recorded from Yukon Territory and British Columbia to NF (Bousquet et al. 2013). It breeds in the phloem of *Picea* spp. (Bright 1976).

**Specimens examined**: Labrador: Labrador City ski hill, 18 July 1981, M. Colbo and D. Larson (5, DLC, Accession Nos 17-20717 to 17-20721; 1, NFRC, Accession No. NFRC-2022-13362; determined by DWL and all DLC specimens but 17-20718 confirmed by DNA barcodes – BOLD: CCDB-28537-B10, -B12, -C01, -C02).

***Ipspini* (Say, 1826)**

This Nearctic species is one of the most common and widely distributed *Ips* in North America, and in Canada it was previously recorded from Yukon Territory and British Columbia to NF (Bousquet et al. 2013). Here we report it for the first time from LB. Larvae feed in the phloem and cambium of the boles and large branches of weakened or recently dead *Pinus* (rarely *Picea*) (Bright 1976). Only jack pine, *Pinusbanksiana* Lamb., occurs in LB so undoubtedly this is the host.

**Specimens examined**: Labrador: Cartwright Highway, 52.9187°N, 60.2360°W, 5 August 2008, ex log deck, G. Pohl and D. Langor (4, DLC, Accession Nos 17-20752 to 17-20755; 3, NFRC, Accession Nos NFRC-2022-13367 to 2022-13369; determined by DWL and all DLC specimens except for 17-20754 confirmed by DNA barcodes – BOLD: CCDB-28537-C03, -C04, -C06).

**Scolytinae: Phloeotribini**

***Phloeotribuspiceae* Swaine, 1911**

This Nearctic species is recorded for the first time from NL based on a single specimen from NF. In Canada, it has been recorded from Yukon Territory and British Columbia to Nova Scotia (Bousquet et al. 2013). Larvae breed in the phloem of *Picea* spp. (Bright 1976).

**Specimens examined**: Newfoundland: Corner Brook, near Loggers School Road, 28 June to 13 July 1994, ex pitfall trap in 60 year-old fir-*Dryopterus* forest (1, NFRC, Accession No. NFRC-2022-13382; determined by DWL).

### ﻿NEMONYCHIDAE

#### Cimberidinae: Cimberidini

***Cimberispilosa* (LeConte, 1876)**

This is the first record of this family and Nearctic species from NL, based on material from NF. Adults have been collected on *Pinusbanksiana*, *P.palustris* Mill., *P.taeda* L., and *P.virginiana* Mill., and larvae on *P.virginiana* (Kuschel 1989).

**Specimens examined**: Newfoundland: 1.5 km E of Long Harbour on Route 1, 28 June 1973, FIS 73-1-0268(03), *Abiesbalsamea* (1, CFS, determined by DWL).

## ﻿Notes on other species

### ﻿ATTELABIDAE

***Temnoceruscyanellus* (LeConte, 1876)** [Rhynchitinae]

Blatchley and Leng (1916) report this Nearctic species from NF but give no specific collection localities nor a source of the record. Additionally, Hamilton (1971) gives no NL localities for this species, nor have we seen specimens or verified records from NL. This species is widely distributed in Canada, from Northwest Territories to Nova Scotia, and the plant species with which it is associated – *Betula*, *Quercus* and *Salix* (Bright, 1993) – occur in NL. Nonetheless, we consider the record from NF as questionable.

### ﻿CURCULIONIDAE

***Ceutorhynchusquerceti* (Gyllenhal, 1813)** [Ceutorhynchinae]

This Nearctic species is recorded from NF (as *C.pusio* Mannerheim) by O’Brien and Wibmer (1982), and this record was reiterated by McNamara (1991a) and Bousquet et al. (2013). Dietz (1896) did not record it from NL. To date we have not seen verified locality records or specimens of this species from NL, although we believe the species is likely on the island of NF.

***Dietzellazimmermanni* (Gyllenhal, 1837)** [Ceutorhynchinae]

This Nearctic species is recorded from NF by O’Brien and Wibmer (1982), a record reiterated by McNamara (1991a) and Bousquet et al. (2013); however, we have seen no specimens or verified locality records. Dietz (1896) did not record this species from NL. The species is currently recorded from British Columbia to New Brunswick (Bousquet et al. 2013) so it is possible that it occurs in NF. However, we consider the record as questionable.

***Phytobiusleucogaster* (Marsham, 1892)** [Ceutorhynchinae]

This Holarctic species is recorded from NF by O’Brien and Wibmer (1982), a record also reported by McNamara (1991a) and Bousquet et al. (2013), but we have seen no specimens or verified locality records. It was not reported from NL by Dietz (1896). The species is recorded from Yukon Territory and from British Columbia to Quebec. Given the lack of verified records of this species east of Quebec, we opt to remove it from the species list for NL.

***Isochnussequensi* (Stierlin, 1894)** [Curculioninae]

This Palearctic species was first recorded in North America under the name *Isochnuspopulicola* (Silfverberg), a junior synonym of *I.sequensi*. Anderson (1989) and McNamara (1991a) did not record this species from NF, but Sweeney et al. (2012) and Bousquet et al. (2013) did record it from the province. There do not appear to be any published locality records for NL. This weevil is widely distributed across the island of NF.

**Specimens examined**: Newfoundland (determinations by RSA): Bay D’Espoir, 14 July 1985, Lloyd Hollet [1, DLC, Accession No. 17-24845]; Blue Pond Park, 48.783°N, 58.087°W, 212 m, sweep of lakeshore vegetation, 25 June 2010, David Langor [2 DLC, Accession Nos 12-117 and 17-17848]; Cape St. George, 48.934°N, 59.263°W, coastal tundra, 11 July 2008, Goulet, Boudreault & Badiss [6, DLC, Accession Nos 17-17841 to 17-17846]; Cormack, 12 June 1979, Larson & Swales [8, DLC, Accession Nos 12-95, 17-17769 to 17-17776]; Corner Brook, mid July 2004, poplar, E. Burke [2, DLC, Accession Nos 12-104 and 17-17761]; Corner Brook, 48.975°N, 57.9°W, 10 July 2008, fallow field, sweep sample #11, Goulet, Boudreault & Badiss [2, DLC, Accession Nos 12-113 and 17-17816]; Corner Brook, 17 Sept. 2002, willow, H. Crummey [1, DLC, Accession No. 17-17840]; Deer Lake, 49.2°N, 57.46°W, 12 July 2008, fallow field, sweep sample #5, Goulet, Boudreault & Badiss [5, DLC, Accession Nos 12-116, 17-17819 to 17-178222]; Deer Lake shoreline, near Pynn’s Brook, 49.0917°N, 57.5460W, 25 June 2011, detritus, David Langor & Greg Pohl [1, DLC, Accession No. 17-17847]; Grandaddy’s Brook, NE of Millville, 16 June 1979, Larson & Swales [1, DLC, Accession No. 12-100]; Gros Morne National Park, near Sally’s Cove, 20 June 1979, near stream, Larson & Swales [8, DLC, Accession Nos 12-96, 17-17762 to 17-17768]; junction of TCH and Codroy River, 11 June 1979, small stream, Larson & Swales [2, DLC, Accession Nos 12-98 and 17-17808]; junction of Hwy. 430 & Upper Humber River, 12 June 1979, Larson & Swales [1, DLC, Accession No. 12-105]; Lomond, 49.4619°N, 57.7610°W, 3 m, 6 July 2006, sweep sample, D. Langor [7, DLC, Accession Nos 12-108, 17-17802 to 17-17807; 2, NFRC, Accession Nos NFRC-2022-07149 and 2022-07150]; Noel Paul River, 48.561°N, 58.491°W, 50 m, 22 June 2010, vegetation, sweep, David Langor [2, DLC, Accession Nos 12-120 and 17-17827; 1, NFRC, Accession No. NFRC-2022-07148]; Pasadena, 23 July 1984, D. Langor [1, DLC, Accession No. 12-101]; Ibid. 10 August 1982 [4, DLC, 12-94, 17-17755 to 17-17757]; Ibid., 23 July 1984 [2, DLC, Accession Nos 17-17797 and 17-17798]; Pasadena, 5 July 1993, malaise trap, L. Perry [1, DLC, Accession No. 17-24846]; Pasadena Beach, 49.022°N, 57.608°W, 31 m, 25 June 2010, vegetation, sweep, D. Langor [7, DLC, Accession Nos 12-121, 17-17828, 17-17829, 17-17831, 17-17832, 17-17834; 2, NFRC, Accession Nos NFRC-2022-07151 and 2022-07152]; Port Saunders, 50.65°N, 57.26°W, 16 July 2008, fallow field, sweep sample #10, Goulet, Boudreault & Badiss [3, DLC, Accession Nos 12-111, 17-17811, 17-17812]; Portugal Cove, Nov. 2000, ex. willow, David Larson [2, DLC, Accession No. 17-17760; 1, NFRC, Accession No. NFRC-2022-07153]; Pynn’s Brook, 13 June 1979, Larson & Swales [3, DLC, Accession Nos 12-99, 17-17809, 17-17810]; Sandy Lake, 49.2874°N, 56.8743W, 317 m, 11 August 2008, shoreline and vegetation, sweep, D. Langor [1, DLC, Accession No. 12-107]; Sandy Lake, 49.288°N, 56.871°W, 100 m, 20 June 2010, *Salix*, sweep, David Langor [1, DLC, Accession No. 12-122]; South Branch, 47.92°N, 59.03°W, 10 July 2008, fallow field, sweep sample #1, Goulet, Boudreault & Badiss [4, DLC, Accession Nos 12-112, 17-17813 to 17-17815]; St. Andrew’s, 47.793°N, 59.232°W, 17 June 2004, sweep of field, Henri Goulet [3, DLC, Accession Nos 12-110, 17-17758, 17-17759]; St. Andrew’s, 47.79°N, 59.235°W, 15 m, 18 July 2008, fallow field, sweep sample #12, Goulet, Boudreault & Badiss [2, DLC, Accession Nos 12-119 and 17-17823]; St. David’s, 48.2°N, 58.6°W, 10 July 2008, fallow field, sweep sample #2, Goulet, Boudreault & Badiss [3, DLC, Accession Nos 12-115, 17-17817, 17-17818]; St. John’s, 12 Sept. 1996 [1, DLC, Accession No. 12-97]; St. John’s, Newfoundland Drive, 47.6010°N, 52.712°W, 83 m, 2 July 2010, vegetation, sweep, D. Langor [3, DLC, Accession Nos 12-119, 17-17824, 17-17825]; Ibid., 20 June 2009 [4, DLC, Accession Nos 12-123, 17-17836 to 17-17838]; St. John’s, Oxen Pond Botanic Park, 23 Aug. to 4 Sept. 1999, David Larson [18, DLC, Accession Nos 12-102, 17-17780 to 17-17796]; St. John’s, Quidi Vidi Lake outlet, 47.5841°N, 52.6799°W, 15 m, 20 June 2009, sweep, D. Langor [1, DLC, Accession No. 12-114].

***Tachyergessalicis* (Linnaeus, 1758)** [Curculioninae]

Although O’Brien and Wibmer (1982) recorded this Holarctic species from NF, Anderson (1989) was unable to substantiate the record. To date, we have not seen specimens or verified locality records of this species from the province. However, the species has a transcontinental distribution and occurs from British Columbia to Nova Scotia (Bousquet et al. 2013), so it possibly occurs in NF.

***Phyxelisrigidus* (Say, 1832)** [Entiminae]

McNamara (1991a) first reported this Nearctic species from NL; however, neither Bright and Bouchard (2008) or Anderson (2018) could confirm the record, but it is possible that the species occurs in the province based on its wide distribution in Canada. Still no specimens or verified locality records of this species from NL are known so its presence there cannot be verified and we opt to remove it from the species list for the province.

***Magdalishispoides* LeConte, 1876** [Mesoptiliinae]

This Nearctic species was recorded from NF by O’Brien and Wibmer (1982) but we have found no specimens or verified locality records of this species from the province. The species is common and has a wide distribution, from British Columbia to New Brunswick in Canada (Bousquet et al. 2013), therefore it possibly occurs in NL.

***Conotrachelusnenuphar* (Herbst, 1797)** [Molytinae]

This Nearctic species was recorded from NF by O’Brien and Wibmer (1982) but we have not seen specimens or verified locality records. The species is recorded from British Columbia to Prince Edward Island (Bousquet et al. 2013), so it possibly occurs in NL.

***Hylobiuspinicola* (Couper, 1864)** [Molytinae]

This Nearctic species is widespread in NF and was also recorded from LB by Bousquet et al. (2013); however, we have not seen specimens or verified locality records from LB. Nonetheless it is highly likely the species occurs there as it is widely distributed in Canada and its hosts occur in LB.

***Lepyrusnordenskioeldialternans* Casey, 1895** [Molytinae]

This Nearctic subspecies was reported from only LB by Van Dyke (1928), a record also reported by McNamara (1991a) and Bousquet et al. (2013); however, we have not seen specimens or verified locality records from LB. The two other subspecies in Canada are reported from Yukon to Saskatchewan (Bousquet et al. 2013). It seems unlikely the species (and subspecies) is present in LB so we remove it from the species list for NL.

***Pachylobiuspicivorus* (Germar, 1824)** [Molytinae]

This Nearctic species was reported from LB by O’Brien and Wibmer (1982), but we have not seen specimens or verified locality records. The only other records from Canada are from Ontario, but the species is widespread in the USA. It seems unlikely that the record from LB is valid, so we hereby remove it from the species list for NL.

***Trachodeshispidus* (Linnaeus, 1758)** [Molytinae]

This Palearctic species was recorded from NF by O’Brien and Wibmer (1982), and this is the only North American jurisdiction from which this species is recorded. We have not seen specimens or verified records from NL, but there is a specimen from the French island of St. Pierre, near the south coast of NF, collected in 1951 (CNC). It is possible that this specimen was the source of the putative record for NF. As the NF record cannot be confirmed, and may simply represent a mistaken attribution of St. Pierre as a part of NF, we opt to remove this species from the list for NL and Canada. However, the species is clearly recorded from North America, although it is uncertain whether it is still established in St. Pierre.

***Pityophthoruscascoensis* Blackman, 1928** [Scolytinae]

This Nearctic species was not recorded from NL by Bright (1981) but was recorded from “Newfoundland” by Wood (1982). We have not seen specimens or verified locality records from NL. The species is also recorded in Canada from Alberta, Northwest Territories and Ontario (Bousquet et al. 2013). Since there is a Newfoundland, New Jersey it is possible the record is from there rather than from NL. We opt to remove this species from the checklist for NL.

***Pityophthoruspuberulus* (LeConte, 1868)** [Scolytinae]

This Nearctic species was not recorded from NL by Bright (1981) or Wood (1982) but was recorded from NF by McNamara (1991b). We have not seen specimens or verified locality records from NL. The species is recorded from Ontario to Nova Scotia so its presence in NF is possible. Hence, we consider the presence of this species in NF to be questionable.

***Dendroctonuspunctatus* LeConte, 1868** [Scolytinae]

This Nearctic species was recorded from NF by McNamara (1991b), but we have seen no specimens or verified locality records. In Canada, it is distributed from British Columbia to New Brunswick (Bousquet et al. 2013), so its presence in NL is highly likely, especially given its hosts (mainly *Picea* spp. and occasionally *Pinus* and *Larix*; Bright 1976) are widespread in the province.

***Dendroctonussimplex* LeConte, 1868** [Scolytinae]

This Nearctic species is widespread in NF where it sometimes causes outbreaks in *Larixlaricina* (Du Roi) K. Koch (Pinaceae) (Langor and Raske 1989). It was reported from LB by McNamara (1991b), but we have not seen specimens or verified locality records from there. Nonetheless, most likely it occurs in LB as its host is abundant there.

***Dendroctonusvalens* LeConte, 1857** [Scolytinae]

Bright (1976) recorded this Nearctic species from NF; however, we have seen no specimens or verified locality records from there. One specimen in the CNC, where Bright worked, is from Newfoundland, New Jersey, and this may have been mistakenly recorded by him as from NL. Thus, we remove this species from the list for NL; however, because of its wide distribution in Canada (Bousquet et al. 2013) and the presence of its hosts (mainly *Pinus*; Bright 1976) in NF, it is possible it occurs there.

***Ipsperturbatus* (Eichhoff, 1869)** [Scolytinae]

This Nearctic species was recorded from LB by McNamara (1991b), but we have not seen specimens or verified records. The species has a wide distribution and its host (*Picea*; Bright 1976) occurs in LB so it seems likely the species is there and in NF as well.

***Pityokteinessparsus* (LeConte, 1868)** [Scolytinae]

This Nearctic species was reported from NF and LB by McNamara (1991b), but we have not seen specimens or verified records from LB. Nonetheless, given its wide distribution in Canada (Bousquet et al. 2013) and presence of its host (*Abies*; Bright 1976) in LB, we expect it occurs there.

***Scolytuspiceae* (Swaine, 1910)** [Scolytinae]

This Nearctic species was reported from NF and LB by McNamara (1991b), but we have not seen specimens or verified records from LB. Nonetheless, given its wide distribution in Canada (Bousquet et al. 2013) and presence of its hosts (mainly *Picea*; Bright 1976) in LB, we expect it occurs there.

## ﻿Synopsis

With the addition of 30 species and removal of seven unsubstantiated species records from the province’s fauna, currently 134 curculionoid species are recorded from NL as a whole, 126 from NF and 36 from LB (Table 1). We could not locate specimens for nine species previously reported by Bousquet et al. (2013) for either NF or LB; however, we opted to retain these records for now because their presence in the Maritime provinces and the availability of suitable host species and climate in NL suggests that these records could be valid, with specimens residing in collections other than those examined for this study.

**Table 1.** List of the species of Curculionoidea from the province of Newfoundland and Labrador. Species preceded by an asterisk (*) were documented from the province by the Fennoscandinavian expeditions of 1949 and 1951. A collection event is a unique combination of locality and date where one or more specimens were collected, labelled, and deposited in a public repository. The dataset was compiled using more than 5130 observed and validated specimen records and was the source of information to identify the year and locality of earliest detection for non-native species.

Taxon ^1^	Distribution ^2^	No. collection events ^3^	Year of earliest [most recent] NL records, and site of first provincial record for non-native species only ^4^
Nemonychidae			
Cimberidinae			
Cimberidini			
*Cimberispilosa* (LeConte, 1876)	ON QC NB **NF**^NPR^	1	1973 [1973]
Anthribidae			
Anthribinae			
Tropiderini			
*Gonotropisdorsalis (Thunberg, 1796) ^H^	YT NT AB SK MB QC NB NF	2	1949 [1984]
Attelabidae			
Rhynchitinae			
Auletini			
*Auletobiuscassandrae (LeConte, 1876)	SK ON QC NB NS PE NF	5	1949 [2010]
Rhynchitini			
*Temnoceruscyanellus* (LeConte, 1876)	NT AB SK MB ON QC NB NS NF^?^	0	NA
Brentidae			
Apioninae			
Apionini			
*Betulapionsimilewalshii (J.B. Smith, 1884)	BC AB SK MB ON QC NB NS **LB**NF	9//1	1949/1978 [1975/2008]
*Perapioncurtirostre* (Germar, 1817) ^I^	QC NB NS **NF**^NPR^	18	1999, St. John’s, [2014]
Curculionidae			
Brachycerinae			
Erirhinini			
*Notarisaethiops (Fabricius, 1792) ^H^	YK NT BC AB SK MB ON QC NB NS LBNF	38//6	1949/2004 [2011/2009]
*Notarispuncticollis (LeConte, 1876)	NT BC AB SK MB ON QC NB NS PE NF	2	**1951 [1951**]
*Tournotarisbimaculata (Fabricius, 1787) ^H^	YK NT BC AB SK MB ON QC NB NS NF	10	1949 [2003]
Curculioninae			
Acalyptini			
*Acalyptuscarpini* (Fabricius, 1792)	YT NT BC AB SK MB ON QC NB NS **LB**^NPR^	1	2008 [2008]
Anthonomini			
* Anthonomus (Anthonomus) corvulus LeConte, 1876	BC AB SK MB ON QC NB NS PE NF	5	1949 [2010]
Anthonomus (Anthonomus) lecontei Burke, 1975	BC AB SK MB ON QC NB NS PE **NF**^NPR^	1	2006 [2006]
Anthonomus (Anthonomus) signatus Say, 1832	BC AB SK MB ON QC NB NS PE NF	1	1977 [2010]
Anthonomus (Paranthonomus) rubidus LeConte, 1876	BC ON QC **NF**^NPR^	2	1982 [1982]
*Anthonomus (Tachypterellus) quadrigibbus Say*, 1832	BC AB SK MB ON QC NB NS **NF**^NPR^	15	2012 [2012]
Ellescini			
*Dorytomuslaticollis* LeConte, 1876	BC AB SK MB ON QC NB NS **LB**^NPR^	1	2009 [2009]
*Dorytomusparvicollis* Casey, 1892	BC AB SK MB ON QC NB NS **NF**^NPR^	1	**1968 [1968**]
*Dorytomusrufulus* (Mannerheim, 1853) ^H^	YT NT BC AB SK ON QC NS **LB**^NPR^	1	1981 [1981]
*Dorytomusvagenotatus Casey, 1892	YT NT BC AB SK ON QC NB **LB**NF	2//2	1949/2008 [1966/2008]
*Ellescusephippiatus (Say, 1832)	YT BC AB SK ON QC NB NF	1	**1949 [1949**]
Mecinini			
*Mecinuspascuorum* (Gyllenhal, 1813) ^I^	BC ON QC NS PE **NF**^NPR^	1	2022, Frenchmans Cove, [2022]
*Rhinusaantirrhini* (Paykull, 1800) ^I^	BC AB SK MB ON QC NB NS PE NF	24	1953, Topsail [2006]
Rhamphini			
*Isochnusrufipes* (LeConte, 1876)	BC AB SK MB ON QC NB NS NF	1	1979 [1979]
*Isochnussequensi* (Stierlin, 1894) ^I^	ON QC NB NS PE NF	34	1979, 6 sites in W. NF, [2012]
*Orchestespallicornis* Say, 1832	BC AB MB ON QC NB NS **LB**NF^?^	0//1	NA/2008 [NA/2008]
*Orchestestestaceus (O.F. Müller, 1776) ^H^	BC AB SK MB ON QC NB NS NF	20	1949 [2010]
*Tachyergesniger* (Horn, 1873)	YT NT BC AB SK MB ON QC NB PE LB	2	2008 [2009]
*Tachyergessalicis* (Linnaeus, 1758) ^H^	NT BC AB SK MB ON QC NB NS NF^?^	0	NA
Styphlini			
*Orthochaetessetiger* ([Beck, 1817]) ^I^	**BC NF** ^NCR^	2	1995, St. John’s, [1999]
Tychiini			
*Tychiuspicirostris* (Fabricius, 1787) ^I^	BC AB SK MB ON QC NB NS PE **LB**NF	24//3	1965, St. John’s, [2010/2009]
*Tychiusstephensi* Schönherr, 1836 ^I^	BC AB SK ON QC NB NS PE **NF**^NPR^	13	1995, Paddy’s Pond, [2010]
Ceutorhynchinae			
Ceutorhynchini			
*Amalusscortillum* (Herbst, 1795) ^I^	BC AB SK MB ON QC NB NS NF	1	**1965, St. John’s, [1965**]
*Ceutorhynchusamericanus* Buchanan, 1937	YT NT BC AB SK MB ON QC NB NS **NF**^NPR^	1	2005 [2005]
*Ceutorhynchushamiltoni Dietz, 1896	QC NB NS PE NF	16	1949 [2010]
*Ceutorhynchusomissus Fall, 1917	AB SK MB ON QC NB NS **NF**^NPR^	1	**1949 [1949**]
*Ceutorhynchusoregonensis* Dietz, 1896	YT BC AB MB ON QC NS **NF**^NPR^	1	2008 [2008]
*Ceutorhynchusquerceti* (Gyllenhal, 1813) ^H^	NT BC AB MB QC NB NS PE NF^?^	0	NA
*Ceutorhynchussemirufus LeConte, 1876	AB ON QC NB NF	2	1951 [1977]
*Ceutorhynchustyphae (Herbst, 1795) ^I^	ON QC NB NS NF	10	1949, Harmon Field, [1977]
*Glocianuspunctiger (C.R. Sahlberg, 1835) ^I^	YT BC AB SK MB ON QC NB NS PE NF	27	1949, widespread, [2008]
Cnemogonini			
*Auleutesepilobii (Paykull, 1800) ^H^	YT NT BC AB SK MB ON QC NB NS PE NF	17	1949 [2008]
*Cnemogonuslecontei* Dietz, 1896	YT NT BC AB MB ON QC NB **LB**^NPR^	2	1982 [2008]
*Dietzellazimmermanni* (Gyllenhal, 1837)	BC SK ON QC NB NF^?^	0	NA
*Perigasterliturata (Dietz, 1896)	YT ON QC NB NS PE NF	2	1949 [1973]
Phytobiini			
*Pelenomusfuliginosus* (Dietz, 1896)	BC AB ON QC NB **LB**^NPR^	2	2002 [2002]
*Rhinoncusbruchoides* (Herbst, 1784) ^I^	QC NS **NF**^NPR^	1	2008, Logy Bay, [2008]
*Rhinoncusleucostigma (Marsham, 1802) ^I^	BC ON QC NB NS PE NF	16	1949, St. John’s, [2001]
*Rhinoncuspericarpius (Linnaeus, 1758) ^I^	BC ON QC NB NS PE **LB**NF	28//3	1949/1980, widespread, [2005/2008]
Scleropterini			
*Prorutidosomadecipiens* (LeConte, 1876)	YT BC AB SK MB ON QC PE **LBNF**^NPR^	3//1	2003/2002 [2003/2002]
Cossoninae			
Cossonini			
*Cossonusamericanus Buchanan, 1936	SK QC NB NS NF	5	1949 [1995]
Pentarthrini			
*Euophryumconfine* (Broun, 1880) ^I^	NF	9	1978, St. John’s, [2002]
*Pentarthrumhuttoni* Wollaston, 1854 ^I^	QC BF	6	1978, St. John’s, [1994]
Rhyncolini			
*Carphonotustestaceus Casey, 1892	BC AB SK ON QC NB NS PE NF	7	1949 [1992]
*Rhyncolusbrunneus Mannerheim, 1843	YT NT BC AB SK MB ON QC NB NS PE **LB**NF	23//1	1949 [2011]
*Rhyncolusmacrops* Buchanan 1946	BC ON QC NB NS **NF**^NPR^	1	2011 [2011]
Cryptorhynchinae			
Cryptorhynchini			
*Cryptorhynchuslapathi* (Linnaeus, 1758) ^H^	BC AB SK MB ON QC NB NS PE NF	23	1958 [2002]
Cyclominae			
Listroderini			
*Listronotushumilis (Gyllenhal, 1834)	BC QC NB NF	8	**1949 [1964**]
Dryophthorinae			
Rhynchophorini			
*Sitophilusgranarius* (Linnaeus, 1758) ^I^	BC AB SK MB ON QC NB NS PE NF	9	1974, St. John’s [1995]
*Sitophilusoryzae* (Linnaeus, 1763) ^I^	BC AB SK ON QC NB NS PE NF	9	1966, Port aux Basques, [2000]
*Sitophiluszeamais* Motschulsky, 1855 ^I^	MB ON QC **NF**^NPR^	1	1985, St. John’s, [1985]
Entiminae			
Brachyderini			
*Strophosomamelanogrammum (Forster, 1771) ^I^	BC ON QC NS PE NF	86	1949, widespread, [2012]
Cneorhinini			
*Philopedonplagiatum (Schaller, 1783) ^I^	ON QC NB NS PE NF	23	1949, Port aux Basques, [2010]
Geonemini			
*Barynotusobscurus (Fabricius, 1775) ^I^	BC QC NB NS PE NF	27	1949, widespread, [2004]
*Barynotusschoenherri (Zetterstedt, 1838) ^I^	QC NB NS PE NF	49	1949, widespread, [2012]
Hormorini			
*Hormorusundulatus (Uhler, 1856)	AB MB ON QC NB NS PE NF	2	1949 [2013]
Otiorhynchini			
*Otiorhynchusdesertus Rosenhauer, 1847 ^I^	NF	3	1949, Cape Broyle, [1997]
*Otiorhynchusligneus (Olivier, 1807) ^I^	QC NB NS PE NF	11	1949, widespread, [1989]
*Otiorhynchusovatus (Linnaeus, 1758) ^I^	YT NT BC AB SK MB ON QC NB NS PE LBNF	145//6	1949, widespread, [2003/2008]
*Otiorhynchusporcatus* (Herbst, 1795) ^I^	ON QC NC NF	8	1965, St. John’s, [1992]
*Otiorhynchusrugifrons (Gyllenhal, 1813) ^I^	QC NB NS NF	58	1949, widespread, [2011]
*Otiorhynchussingularis (Linnaeus, 1767) ^I^	BC ON QC NB NS PE NF	72	1944, St. John’s, [2012]
*Otiorhynchussulcatus (Fabricius, 1775) ^I^	BC AB SK MB ON QC NB NS PE NF	240	1949, widespread, [2011]
Peritelini			
*Nemocesteshorni* Van Dyke, 1936	BC SK ON QC NB NS NF	4	1966 [1983]
Phyllobiini			
*Phyllobiusoblongus* (Linnaeus, 1758) ^I^	BC ON QC NB NS PE **NF**^NPR^	3	2010, Corner Brook, [2011]
Polydrusini			
*Pachyrhinuselegans* (Couper, 1865)	BC AB SK AB QC NB NS **NF**^NPR^	2	2022, western NL, [2022]
*Polydrususcervinus* (Linnaeus, 1758) ^I^	QC NS PE **NF**^NPR^	1	2010, Tompkins, [2010]
*Polydrususformosus* (Mayer, 1779) ^I^	BC ON QC NB NS PE **NF**^NPR^	12	2002, Corner Brook, [2013]
Sciaphilini			
*Barypeithespellucidus (Boheman, 1834) ^I^	BC MB ON QC NB NS PE NF	56	1949, widespread, [2010]
*Brachysomusechinatus (Bonsdorff, 1785) ^I^	QC NF	2	**1949, widespread, [1949**]
*Sciaphilusasperatus (Bonsdorff, 1785) ^I^	BC AB MB ON QC NB NS PE NF	69	1949, widespread, [2011]
Sitonini			
*Sitonacylindricollis* (Fahraeus, 1840) ^I^	YT NT BC AB SK MB ON QC NB NS PE **NF**^NPR^	5	1980, St. John’s, [2008]
*Sitonahispidulus* (Fabricius, 1777) ^I^	NT BC AB SK ON QC NB NS PE NF	5	1979, St. John’s, [1986]
*Sitonalepidus Gyllenhal, 1834 ^I^	BC AB SK MB ON QC NB NS PE NF	86	1949, widespread, [2008]
*Sitonalineellus* (Bonsdorff, 1785) ^H^	YT NT BC AB SK MB ON QC NB NS PE LBNF	11//1	1980/? [2008/?]
Trachyphloeini			
*Romualdiusbifoveolatus ([Beck], 1817) ^I^	BC MB ON QC NB NS PE NF	29	1949, widespread, [2008]
Tropiphorini			
*Tropiphoruselevatus (Herbst, 1795) ^I^	NF	2	**1951, Millertown, [1951**]
*Tropiphorusterricola* (Newman, 1838) ^I^	QC NB NS PE NF	7	1965, St. John’s, [2000]
Hyperinae			
Hyperini			
*Brachypterazoilus (Scopoli, 1763) ^I^	BC ON QC NB NS PE NF	45	1949, widespread, [2000]
*Hyperanigrirostris (Fabricius, 1775) ^I^	BC ON QC NB NS PE NF	69	1949, widespread, [2014]
Lixinae			
Cleonini			
*Stephanocleonusplumbeus LeConte, 1876	AB SK MB QC **LB**NF	4//1	1949/2005 [1993/2005]
Mesoptilinae			
Magdalidini			
*Magdalisalutacea LeConte, 1878	YT NY BC AB SK QC MB **LBNF**^NPR^	1//1	**1949/1948 [1949/1948**]
*Magdalisgentilis* LeConte, 1876	BC **NF**^NPR^	1	**1972 [1972**]
*Magdalishispoides* LeConte, 1876	YT BC AB ON QC NB NF^?^	0	NA
Molytinae			
Conotrachelini			
*Conotrachelusnenuphar* (Herbst, 1797)	BC AB SK MB ON QC NB NS PE NF^?^	0	NA
Hylobiini			
*Hylobiuscongener Dalla Torre, Schenk. & Marsh., 1932	YT NT BC AB SK MB ON QC NB NS PE LBNF	58//18	1949/1942 [2012/2005]
*Hylobiuspinicola (Couper, 1864)	BC AB SK MB ON QC NB NS LB^?^NF	56//0	1949/NA [2011/NA]
*Hylobiuswarreni* Wood, 1957	BC AB SK MB ON QC NB NS **LBNF**^NPR^	15//6	1960/2004 [2012/2009]
Lepyrini			
*Lepyruslabradorensis* Blair, 1933	NT QC LB	2	**1954 [1954**]
*Lepyruspalustris* (Scopoli, 1763) ^H^	NT BC AB SK MB ON QC NB LB**NF**	1//1	2003/2006 [2003/2006]
Molytini			
*Sthereusptinoides* (Germar, 1824) ^H^	BC NB NS NF	8	1965 [1999]
Pissodini			
*Pissodesaffinis Randall, 1838	NT BC AB SK MB ON QC NB NS NF	3	**1945 [1945**]
*Pissodesfiskei Hopkins, 1911	YT BC AB SK MB ON QC NB NS PE NF	2	1953 [1998]
*Pissodesnemorensis Germar, 1824	MB ON QC NB NS PE NF	17	1942 [1989]
*Pissodesrotundatus* LeConte, 1876	YT NT BC AB SK MB ON QC NB NS **LB**NF	5//1	1943/1976 [1989/1976]
*Pissodessimilis Hopkins, 1911	BC AB ON QC NB NS NF	19	1943 [2011]
*Pissodesstriatulus* (Fabricius, 1775)	BC AB SK MB ON QC NB NS PE **LB**NF	18//5	1963/1965 [1988/1986]
Scolytinae			
Corthylini			
*Pityophthorusdentifrons* Blackman, 1922	AB ON QC NB NS PE NF	1	**1970 [1970**]
*Pityophthorusintextus* Swaine, 1917	YT BC AB SK MB ON QC NB NS NF	6	1970 [1982]
*Pityophthorusnitidus Swaine, 1917	YT NT BC AB ON QC NB NS NF	6	1949 [1988]
*Pityophthorusopaculus* LeConte, 1878	YT NT BC AB SK MB ON QC NB NS NF	1	**1970 [1970**]
*Pityophthoruspuberulus* (LeConte, 1868)	ON QC NB NS NF^?^	0	NA
Cryphalini			
* *Cryphalusruficollisruficollis* Hopkins, 1915	YT BC AB MB ON QC NB NS NF	7	1979 [1988]
*Trypophloeusstriatulus* (Mannerheim, 1853)	YT AB MB QC NS NF	1	?
Crypturgini			
*Crypturgusborealis Swaine, 1917	NT BC AB SK MB ON QC NB NS PE NF	4	1949 [1984]
*Crypturguspusillus (Gyllenhal, 1813) ^I^	SK ON QC NB NS PE NF	13	1949, Lomond, [1989]
Dryocoetini			
*Dryocoetesaffaber (Mannerheim, 1852)	YT NT BC AB SK MB ON QC NB NS PE **LB**NF	10//4	1949/1981 [1990/2009]
*Dryocoetesautographus (Ratzeburg, 1837) ^H^	YT NT BC AB SK MB ON QC NB NS PE LBNF	44//5	1949/1982 [2011/2009]
*Dryocoetesbetulae* Hopkins, 1894	BC AB ON QC NB NS NF	15	1970 [2012]
Hylastini			
*Scierusannectans LeConte, 1876	NT BC AB ON QC NB NS PE **LB**NF	3/1	1949/2008 [1984/2008]
Hylurgini			
*Dendroctonuspunctatus* LeConte, 1868	YT NT NU BC AB ON QC NB NF^?^	0	NA
*Dendroctonusrufipennis (Kirby, 1837)	YT NT NU BC AB SK MB ON QC NB NS PE **LB**NF	19//3	1949/2008 [2010/2009]
*Dendroctonussimplex* LeConte, 1868	YT NU BC AB SK MB ON QC NB NS PE LB^?^NF	16/0	1970 [1989]
*Xylechinusamericanus* Blackman, 1922	ON QC NB NS NL	1	2017 [2017]
Ipini			
*Ipsborealis* Swaine, 1911	YT NT BC AB SK MB ON QC NB NS PE **LB**NF	10//1	1970/1981 [1988/1981]
*Ipsperturbatus* (Eichhoff, 1869)	YT NT BC AB SK MB ON QC NB LB^?^	0	NA
*Ipspini* (Say, 1826)	YT NT BC AB SK MB ON QC NB NS PE **LB**NF	6//1	1970/2008 [1984/2008]
*Orthotomicuscaelatus (Eichhoff, 1868)	YT NT BC AB SK MB ON QC NB NS PE NF	3	1949 [1982]
*Pityogeneshopkinsi* Swaine, 1915	SK MB ON QC NB NS NF	4	1970 [1987]
*Pityokteinessparsus* (LeConte, 1868)	AB SK MB ON QC NB NS PE LB^?^NF	10//0	1970/NA [1989/NA]
Phloeotribini			
*Phloeotribuspiceae* Swaine, 1911	YT NT BC AB MB ON QC NB NS NF^NPR^	1	1994 [1994]
Polygraphini			
*Polygraphusrufipennis (Kirby, 1837)	YT NT BC AB SK MB ON QC NB NS PE LBNF	58//6	1949/1981 [2012/2009]
Scolytini			
*Scolytuspiceae (Swaine, 1910)	YT NT BC AB SK MB ON QC NB NS LB^?^NF	5//0	1949/NA [1982/NA]
Xyleborini			
Xyloterini			
*Trypodendronlineatum (Olivier, 1795) ^H^	YT BC AB SK MB ON QC NB NS PE NF	24	1949 [2011]

^1^ * denotes species documented from NL by 1951 (completion of the Fennoscandinavian expeditions); Superscript definitions: H, species with a Holarctic distribution; I, denotes non-native species. ^2^ Jurisdictional acronyms: AB – Alberta, BC – British Columbia, LB – Labrador, MB – Manitoba, NB – New Brunswick, NF – Newfoundland, NS – Nova Scotia, NT – Northwest Territories, NU – Nunavut, O – Ontario, PE – Prince Edward Island, QC – Quebec, SK – Saskatchewan, YT – Yukon. Jurisdictions indicated in bold font denote new records reported herein. Superscript definitions: NPR – new provincial record for NL, NCR – new Canadian record, ? – record for NL or LB that could not be yet verified by the existence of published locality records or observation of a specimen by the authors. ^3^ For entries containing two numbers separated by “/”, the first refers to NF and the second to LB.^4^ Species that have not been captured in NL in the last 50 years are indicated in bold font.

Even with the additional provincial records reported herein, the curculionoid fauna of NL is depauperate compared to adjacent areas of the mainland of Canada. For example, while NL has almost seven times the area of Nova Scotia, it has only half the number of weevil species of its smaller neighbour. Considering only native species, the NL fauna is even more depauperate as only 89 indigenous species, 14 of them with Holarctic distributions, have been documented.

Clearly the province has a high richness of non-native weevils as 45 species (33.6%) of the total NL fauna is comprised of inadvertently introduced species, all from the Palearctic. Only three non-native species are documented from LB (8.3% of the fauna) while all 45 non-native species occur in NF (33.6% of the island fauna). Although other provinces may have more non-native species overall, in terms of proportion of the jurisdictional fauna represented by non-native species, the island of NF is the most ‘Europeanized’ part of the country (Bousquet et al. 2013). Notably, 23 non-native species are Entiminae, representing 85% of the species richness of this subfamily in NF. Larvae of most of the members of this subfamily are free-living in the soil and feed on roots or root nodules of plants (Bright and Bouchard 2008). As 17 of the non-native Entiminae species were already widespread in NF by 1949 (Table 1), and most had widespread distributions, it is highly likely many of these were brought to the island in ballast soil in fishing ships from western Europe (especially southwestern England), which was subsequently deposited on shore (Lindroth 1957). This is seemingly a common means of introduction of many soil dwelling insects and other invertebrates and plants to the island of NF (Lindroth 1957). As for other methods of introduction of non-native weevils, folivorous species may have been imported on plants, species of *Sitophilus* on grains/cereals and Scolytinae and Cossoninae on wood, wood products or dunnage. Some species were likely directly introduced to NL from the Palearctic and others likely spread with human assistance from established populations elsewhere in North America. There were undoubtedly multiple introduction events for some species.

It is now virtually impossible to ascertain exactly when and where currently established non-native species were first introduced to NL. By 1951, when the Fennoscandinavian expeditions to NL were complete, 23 of the 59 curculionoid species documented from the province were non-native (all recorded from NF and none from LB); 16 of those non-native species (including some flightless species) were widely distributed (Table 1) suggesting they had already been present on the island for a long time. From 1961–2010, 3–5 new non-native species were documented from NF per decade, but no new species have been documented from the province since 2010 (Table 1). For the 28 non-native species for which a site of first detection is available (i.e., they were not already widely distributed by the date of first detection), 17 were first documented from St. John’s and vicinity (Table 1). This is not surprising as St. John’s has served as a major port for centuries, and this city was previously suggested as a likely major site of entry for many non-native insects (Langor et al. 2008, 2014). As well, St. John’s likely received much more collecting activity than other parts of the province. Two non-native weevil species were first detected in Port aux Basques, the main ferry port for mainland travel, and two from Corner Brook, the province’s largest city on the west coast (Table 1).

**Figure 2.**
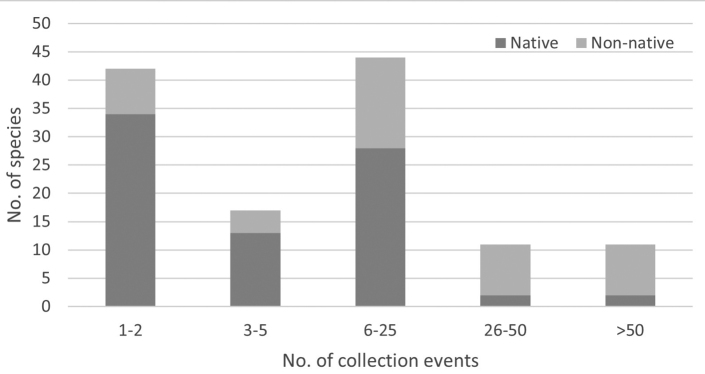
The number of native and non-native species of Curculionoidea in the province of Newfoundland and Labrador, partitioned by the number of collection events for each species. A collection event is a unique combination of locality and date where at least one specimen was collected, labelled, and deposited in a public repository. The dataset was compiled using more than 6400 observed and validated specimen records collected between 1942 and 2022.

Non-native curculionoids in NF are also conspicuous by their relative abundance. According to data from the 1737 specimens in our database that were collected in NL by the conclusion of the Fennoscandinavian expedition in 1951, only two of which were from LB, more than 76% were non-native, representing 25 species. The 415 specimens of native species represented 34 species. Five of the eight most commonly collected species were non-native, and the three most widely and frequently collected species were the non-native *Otiorhynchusovatus* (70 collection events), *O.rugifrons* (33) and *O.sulcatus* (103). By 2022, the date of the most recent record in our database, the dominance of non-native species was even more pronounced as 18 of the 22 most frequently and widely collected weevil species were non-native (Fig. 2; last two columns). During extensive sampling across NF between 2003–2022, sweep net samples were dominated by non-native *Tychius* spp., *Isochnussequensi*, *Glocianuspunctiger*, *Rhinoncus* spp., *Sitonalepidus* and *Polydrususcervinus*, and pitfall traps collected large numbers of *Tournotarisbimaculata* and several species of non-native Entiminae (D. Langor, unpublished data).

With few exceptions, native species of Curculionoidea in NL (especially in NF) are much more uncommonly collected than non-native species. A majority of the 88 native species in the province have only been rarely collected; 34 native species are known from only one or two collection events, and a further 13 are known from 3–5 collection events (Table 1). Fourteen native species have not been collected within the last 50 years (Table 1). These facts give rise to important questions. Does the low incidence of collection of native species reflect low abundance or collection biases? If the abundance is truly low, is this attributable to the high diversity and abundance of non-native species? Unfortunately, our data set is unable to directly address these questions as it represents largely *ad hoc* sampling by many people using a variety of collection methods over more than 75 years. Undoubtedly, other curculionoid species are present in NL and will eventually be documented as sampling continues, especially in western NF and northern LB.
